# Age‐related changes in hippocampal‐dependent synaptic plasticity and memory mediated by p75 neurotrophin receptor

**DOI:** 10.1111/acel.13305

**Published:** 2021-01-15

**Authors:** Lik‐Wei Wong, Yee Song Chong, Wei Lin, Lilian Kisiswa, Eunice Sim, Carlos F. Ibáñez, Sreedharan Sajikumar

**Affiliations:** ^1^ Department of Physiology National University of Singapore Singapore City Singapore; ^2^ Life Sciences Institute Neurobiology Programme National University of Singapore Singapore City Singapore; ^3^ Healthy Longevity Translational Research Programme Yong Loo Lin School of Medicine National University of Singapore Singapore City Singapore; ^4^ Department of Neuroscience Karolinska Institute Stockholm Sweden

**Keywords:** aging, hippocampus, Late‐LTP, p75^NTR^, synaptic capture, synaptic tagging

## Abstract

The plasticity mechanisms in the nervous system that are important for learning and memory are greatly impacted during aging. Notably, hippocampal‐dependent long‐term plasticity and its associative plasticity, such as synaptic tagging and capture (STC), show considerable age‐related decline. The p75 neurotrophin receptor (p75^NTR^) is a negative regulator of structural and functional plasticity in the brain and thus represents a potential candidate to mediate age‐related alterations. However, the mechanisms by which p75^NTR^ affects synaptic plasticity of aged neuronal networks and ultimately contribute to deficits in cognitive function have not been well characterized. Here, we report that mutant mice lacking the p75^NTR^ were resistant to age‐associated changes in long‐term plasticity, associative plasticity, and associative memory. Our study shows that p75^NTR^ is responsible for age‐dependent disruption of hippocampal homeostatic plasticity by modulating several signaling pathways, including BDNF, MAPK, Arc, and RhoA‐ROCK2‐LIMK1‐cofilin. p75^NTR^ may thus represent an important therapeutic target for limiting the age‐related memory and cognitive function deficits.

## Graphical abstract

This cartoon depicts the signaling pathway by p75NTR in mediating synaptic plasticity changes in aging. Aging increases proBDNF without affecting mature BDNF. ProBDNF has been implicated in facilitating LTD. Aging also modulates MAPK pathway by upregulating p38 activity while downregulating ERK1/2 activity. Both p38 and ERK1/2 pathways are important in regulating Arc gene transcription. Aging decreases Arc protein, thus affecting the maintenance of LTP and LTM consolidation through regulation of actin dynamics. In addition, aging increases RhoA level leading to an increase in ROCK2 activity. This reduces both LIMK1 and cofilin phosphorylation. Modulation of cofilin activity is essential for the reorganization of the actin cytoskeleton and influences synaptic plasticity. As a whole, p75NTR is responsible for the age‐mediated disruption of hippocampal homeostatic long‐term plasticity by modulating several signaling pathways, including BDNF, MAPK, Arc, and RhoA‐ROCK2‐LIMK1‐cofilin, leading to deficits in STC and associative memory. Red arrow indicates increases. Green arrow indicates decreases. Orange equals sign indicates no change.

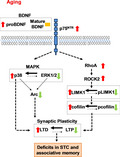

## 1 INTRODUCTION

Aging is thought to play a causal role in the deterioration of cognitive function that can, in part, be explained by changes in synaptic plasticity or cellular alterations that directly affect mechanisms of plasticity (Burke & Barnes, 2006; Mattson & Arumugam, 2018; Shetty et al., 2017). Synaptic plasticity is the ability of synapses to undergo lasting changes in strength in response to activity and is thought to be fundamental for learning and memory. The two most comprehensive models of activity‐dependent bidirectional synaptic plasticity are long‐term potentiation (LTP) and long‐term depression (LTD) (Morris, 2013). The former is a long‐lasting increase in the efficacy and strength of synaptic transmission, while the latter is a long‐lasting decrease in the efficacy and strength of synaptic transmission (Morris, 2013). Interestingly, aberrant LTP and LTD have been associated with age‐related cognitive decline (Bach et al., 1999; Barnes, 1979; Burke & Barnes, 2006). Hippocampus is particularly susceptible to age‐dependent alterations (Burke & Barnes, 2006). Numerous studies have demonstrated that aging reduces hippocampal LTP (Barnes & McNaughton, 1980; Barnes, 1979; Burke & Barnes, 2010; Ojo et al., 2015; Shetty & Sajikumar, 2017), concomitant with memory deficits in aged rodents (Bach et al., 1999; Barnes, 1979; Rapp et al., 1987) as well as in aged humans (Newman & Kaszniak, 2000). Synaptic tagging and capture (STC) is a mechanism of LTP associative plasticity for cellular consolidation that can account for the temporal association of memories (Frey & Morris, 1997). The STC model proposes that glutamatergic activation during LTP induction or memory encoding results in instantaneous local “tagging” of activated synapses. These “synaptic tags” later “capture” the diffusely transported plasticity‐related products (PRPs) synthesized in soma or local dendritic domains (Redondo & Morris, 2011). STC provides a conceptual basis for how short‐term plasticity or memory transforms to long‐term plasticity or memory in a time‐dependent manner and is considered to be an important cellular correlate of associative plasticity and memory formation (Redondo & Morris, 2011). Aging has been shown to impair STC‐related mechanisms (Sharma et al., 2015; Shetty et al., 2017). Contrary to LTP, LTD is enhanced in aging as aged rodents display a heightened susceptibility to induction of LTD and a greater attenuation of previously strengthened synapses (Norris et al., 1996). Taken together, the different changes in hippocampal plasticity that occur during aging make it more difficult for aged synaptic populations to form new memories (Burke & Barnes, 2010; Ojo et al., 2015).

The p75 neurotrophin receptor (p75^NTR^) is a critical regulator of neuronal survival, structure, and function by acting in concert with a collection of ligands and co‐receptors (Dechant & Barde, 2002; Ibáñez & Simi, 2012). This receptor is also implicated in modulating synaptic transmission and plasticity, specifically in the facilitation of LTD (Rösch et al., 2005; Woo et al., 2005). Hence, p75^NTR^ is important in maintaining homeostasis for hippocampal‐dependent synaptic plasticity and efficacy. We have recently reported that p75^NTR^ plays a critical role in sleep deprivation induced plasticity and memory changes in the hippocampus (Wong et al., 2019). Interestingly, p75^NTR^ is expressed in brain regions that are particularly vulnerable in aging, including basal ganglia, cortex, and hippocampus. Increased p75^NTR^ expression has been reported in the aged brain (Costantini et al., 2005) as well as aged‐related disorders, such as Alzheimer's disease (Bachis et al., 2016). However, very little is known about how p75^NTR^ affects plasticity mechanisms of aged neuronal ensembles that ultimately contribute to deficits in cognitive function.

Here, we used genetically modified p75^NTR^ knockout (KO) mice to study the potential role of this receptor in age‐associated homeostatic synaptic plasticity changes in the hippocampus. Our data demonstrate that p75^NTR^ KO mice are resistant to the age‐related changes in long‐term plasticity, associative plasticity, and long‐term memory. We have also investigated the cellular and molecular pathways underlying these effects. These findings highlight the important role of p75^NTR^ in age‐related cognitive decline and suggest that this receptor should be considered as an attractive target for preventing or potentially reversing age‐associated hippocampus alterations during normal aging.

## 2 RESULTS

### 2.1 Deletion of p75^NTR^ prevents age‐associated late‐LTP deficits

**FIGURE 1:**
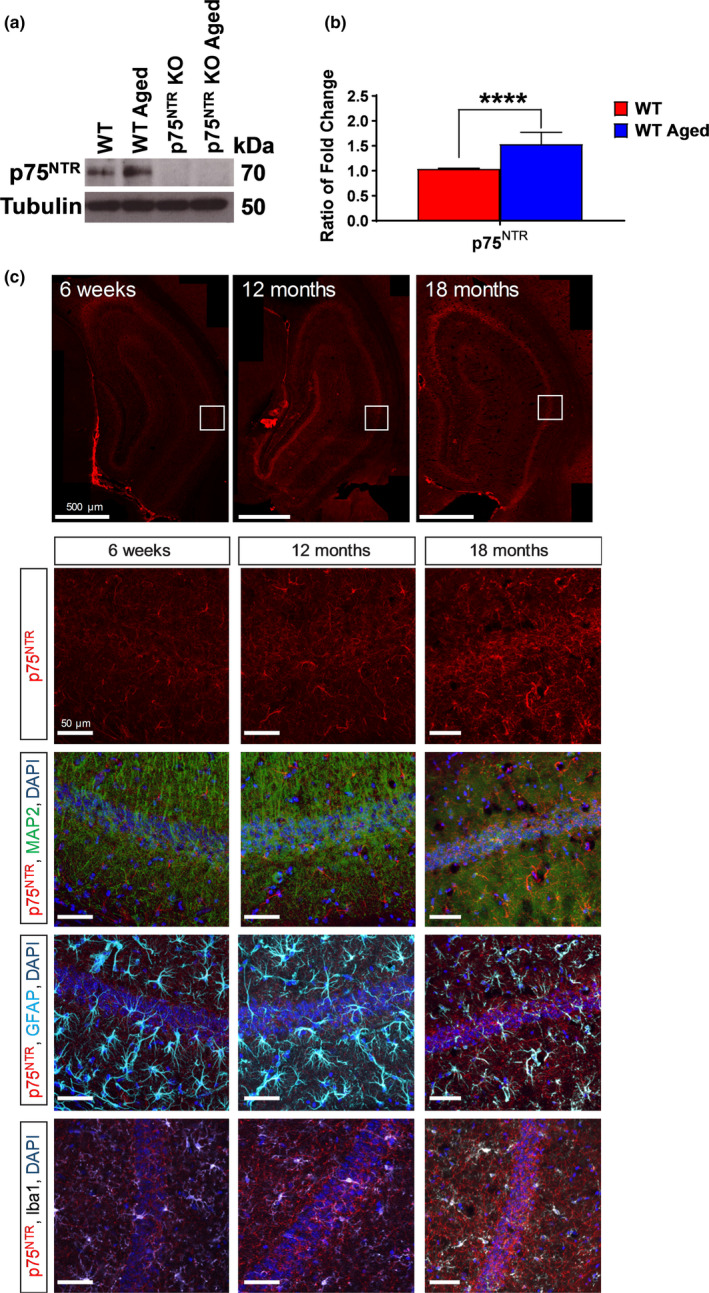
Hippocampal p75^NTR^ protein level increased in aging. (a) Western blot analysis of hippocampal p75^NTR^ level between young adult and aged WT mice. (b) Ratio of fold change from Western blot. The p75^NTR^ protein level was significantly increased (*p* = 0.0001) in aged WT mice compared to young adult WT mice, *N* = 3 for each group. The values of the individual groups were calculated in relation to the control group, while tubulin serves as a loading control. Asterisk indicates significant differences between groups (unpaired *t*‐test, ****p < 0.0001). Error bars indicate ± SEM. (c) Representative photomicrographs of p75^NTR^ (red) with MAP2 (green) or GFAP (light blue) or Iba1 (white), and counterstained with DAPI (dark blue) immunohistochemistry in hippocampal CA1 of 6‐week‐old, 12‐month‐old and 18‐month‐old WT mice

Using Western blotting of hippocampal extracts from aged (22–24 months) and control young adult (5–7 weeks) mice, we found that aging significantly increased (*p* < 0.0001) the expression of p75^NTR^ in the hippocampus of WT mice (Figure 1a, b), similar to previous findings (Costantini et al., 2005; Terry Jr et al., 2011). We have also found that hippocampal p75^NTR^ expression was significantly higher in 12‐month (*p* = 0.0213) and 18‐month (*p* = 0.0009) old WT mice compared to 6‐week‐old WT mice (Figure S1). Furthermore, age‐dependent increase in the expression of p75^NTR^ was found in both the neurons and astrocytes in CA1 area of the hippocampus (Figure 1c). Aging, however, did not affect the expression of p75^NTR^ in microglia in the hippocampal CA1 region (Figure 1c and Figure S2). In addition, our data also demonstrate that hippocampal astrocytic gliosis as shown by GFAP staining was significantly higher (*p* = 0.0136) in aged WT mice compared to aged p75^NTR^ KO mice (Figure S3), suggesting that deletion of p75^NTR^ attenuated age‐dependent change in hippocampal gliosis.

**FIGURE 2:**
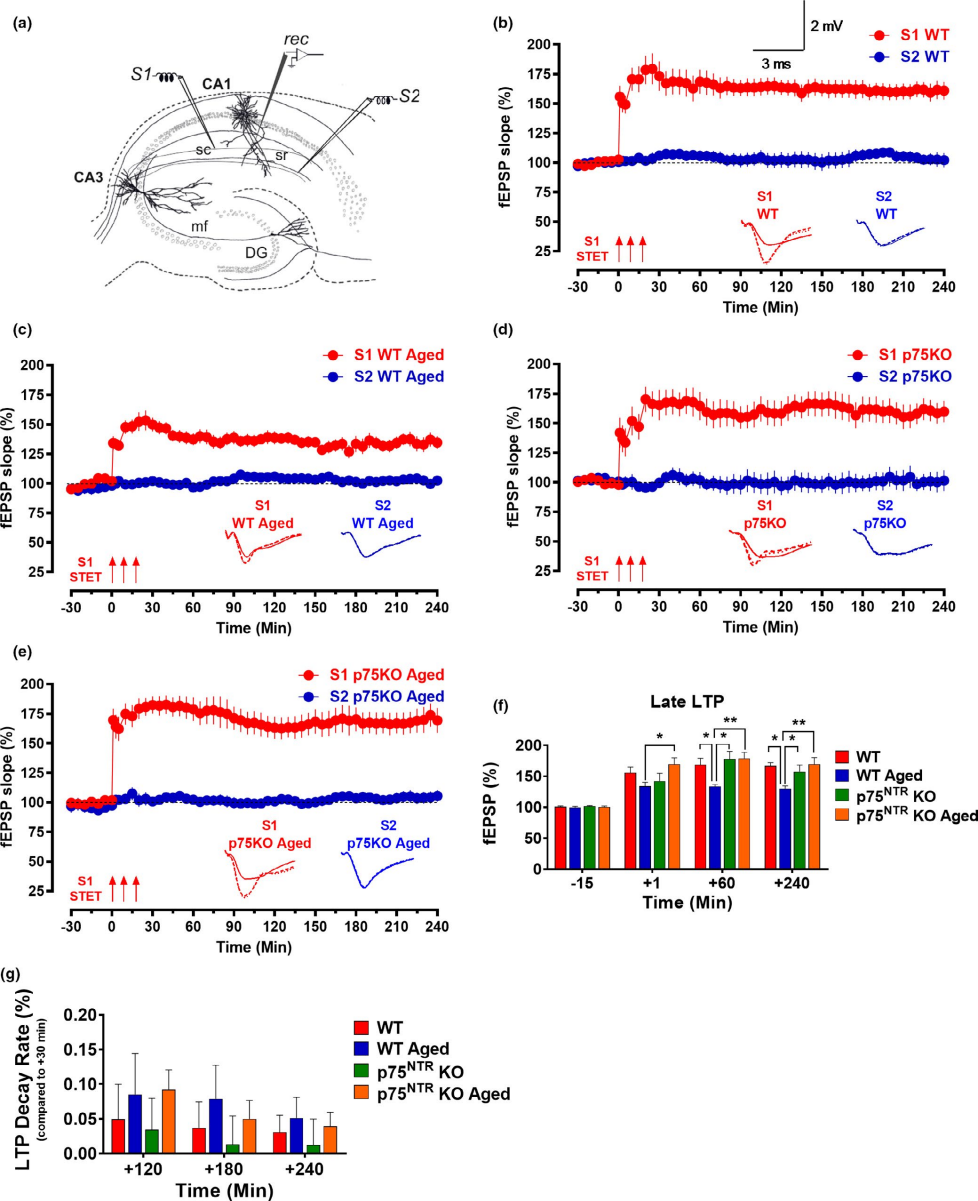
p75^NTR^ KO displayed normal late‐LTP irrespective of age. (a) Schematic representation of a hippocampal slice showing the location of electrodes in the CA1 region. Recording electrode (rec) was positioned onto CA1 apical dendrites flanked by two stimulating electrodes S1 and S2 in the stratum radiatum (sr) to stimulate two independent Schaffer collateral (sc) synaptic inputs to the same neuronal populations. (b) The STET in S1 resulted in a significant potentiation that maintained for 4 h in young adult WT mice (red circles, *N* = 8). (c) The STET in S1 (red circles) resulted in late‐LTP that was significantly maintained toward the end of the recording in aged WT mice (*N* = 8). (d) The STET in S1 resulted in a significant potentiation that maintained for 4 h in young adult p75^NTR^ KO mice (red circles, *N* = 7). (e) STET in S1 (red circles) resulted in a significant potentiation that also maintained for 4 h in aged p75^NTR^ KO mice (*N* = 8). Control potentials from S2 (blue circles) remained stable during the recording period in all experiments. Analog traces represent typical fEPSPs of inputs S1 and S2 15 min before (solid line), 60 min after (dashed line) tetanization, and at the end of the recording (dotted line). Three solid arrows represent STET for the induction of late‐LTP. Scale bars for all the traces vertical: 2 mV; horizontal: 3 ms. Error bars indicate ± SEM. (f) A histogram of mean fEPSP slope values recorded for young adult WT, aged WT, young adult p75^NTR^ KO, and aged p75^NTR^ KO mice at four different time points: 15 min (baseline), +1 min, +60 min, and +240 min after LTP. At +1 min, the mean fEPSP slope value was significantly smaller in aged WT mice compared to aged p75^NTR^ KO (*p* = 0.0345). At +60 min, the mean fEPSP slope value was significantly smaller in aged WT mice compared to young adult WT (*p* = 0.0102), young adult p75^NTR^ KO (*p* = 0.0200) and aged p75^NTR^ KO mice (*p* = 0.0032). At +240 min, the mean fEPSP slope value was significantly smaller in aged WT mice compared to young adult WT (*p* = 0.0275), young adult p75^NTR^ KO (*p* = 0.0369), and aged p75^NTR^ KO mice (*p* = 0.0028). (g) A histogram of LTP decay rate (%) measured for young adult WT, aged WT, young adult p75^NTR^ KO, and aged p75^NTR^ KO mice at three different time points: +120 min, +180 min, and +240 min compared to +30 min after LTP. No significant change in LTP decay rate in aged WT mice compared to young adult WT, young adult p75^NTR^ KO, and aged p75^NTR^ KO mice at all three different time points. Asterisk indicates significant differences between groups (two‐way ANOVA, *p < 0.05, **p < 0.01). Error bars indicate ± SEM

In the first series of electrophysiological experiments, we investigated the induction of LTP by strong tetanization (STET, 3 × 100 Hz, 100 pulses, 10‐min interval, see methods) in Schaffer collateral‐CA1 synapses of hippocampal slices derived from either young adult or aged WT mice. A two‐pathway experimental design was used to study two independent synaptic inputs to the same CA1 pyramidal neuron population (Figure 2a). In young adult WT mice, STET resulted in long‐lasting LTP (red circles). The mean fEPSP slope value immediately after first tetanization was 155.82 ± 9.16% (Wilcox test, *p* = 0.008) and the potentiation was maintained up to 4 h (160.86 ± 1.29%, Wilcox test, *p* = 0.008; *U* test, *p* = 0.0003; Figure 2b). The same stimulation paradigm also resulted in the induction of LTP in aged WT mice with mean fEPSP slope value immediately after first tetanization at 134.17 ± 5.99% (Wilcox test, *p* = 0.008), and the potentiation was maintained for 4 h (134.57 ± 5.33%, Wilcox test, *p* = 0.008; *U* test, *p* = 0.0006; Figure 2c). Four hours after LTP, the magnitude of potentiation was significantly smaller (*p* = 0.0275) in aged WT mice compared to young adult WT mice (Figure 2f). The control synaptic input S2 (blue circles) for both young adult and aged WT mice in late‐LTP experiments remained stable at baseline throughout the entire recording period (Figure 2b,c). In summary, these results show that although normal plasticity can be established in this hippocampal synapse of aged mice, the magnitude of late‐LTP is significantly lower, which is in consistent with our earlier reports (Sharma et al., 2015; Shetty et al., 2017).

**FIGURE 3:**
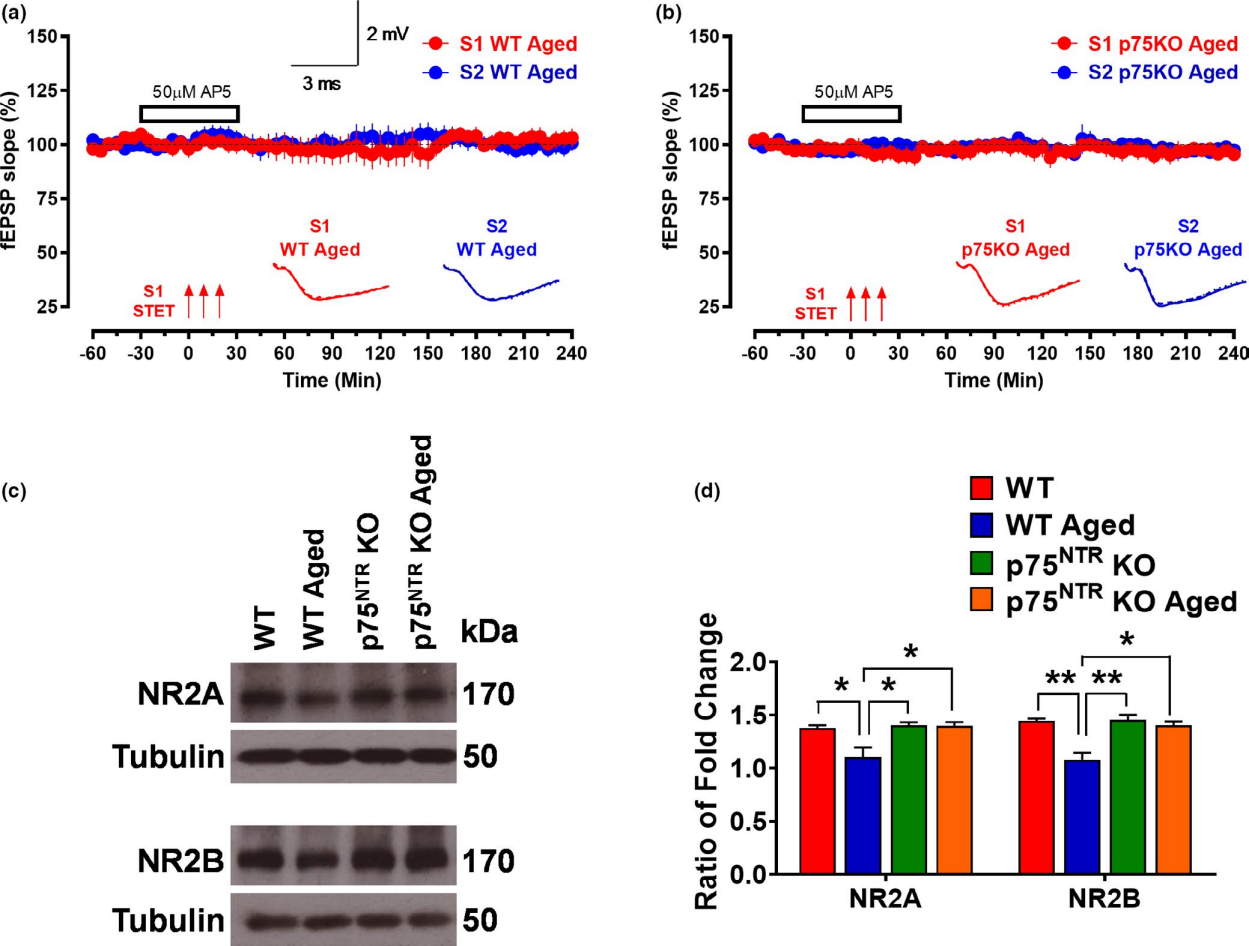
The effect of NMDA receptor antagonist AP5 on LTP in aging and effects of age on NR2A and NR2B levels in the hippocampus. The STET in S1 (red circles) could not induce potentiation in the presence of AP5 (50 μM) in (a) aged WT (*N* = 6) and (b) aged p75^NTR^ KO (*N* = 6) mice. Control potentials from S2 (blue circles) remained stable during the recording period in all experiments. Analog traces represent typical fEPSPs of inputs S1 and S2 15 min before (solid line), 60 min after (dashed line) tetanization, and at the end of the recording (dotted line). Three solid arrows represent STET for the induction of late‐LTP. Scale bars for all the traces vertical: 2 mV; horizontal: 3 ms. Error bars indicate ±SEM. (c) Western blot analysis of hippocampal NR2A and NR2B levels between young adult and aged from WT and p75^NTR^ KO mice. (d) Ratio of fold change from Western blot. The NR2A level was significantly decreased in aged WT mice compared to young adult WT (*p* = 0.0338), young adult p75^NTR^ KO (*p* = 0.0224), and age p75^NTR^ KO (*p* = 0.0243) mice, *N* = 3 for each group. The NR2B level was significantly decreased in aged WT mice compared to young adult WT (*p* = 0.0060), young adult p75^NTR^ KO (*p* = 0.0052) and aged p75^NTR^ KO (*p* = 0.0111) mice, *N* = 3 for each group. The values of the individual groups were calculated in relation to the control group while tubulin serves as a loading control. Asterisk indicates significant differences between groups (two‐way ANOVA, *p < 0.05, **p < 0.01). Error bars indicate ± SEM

**FIGURE 4:**
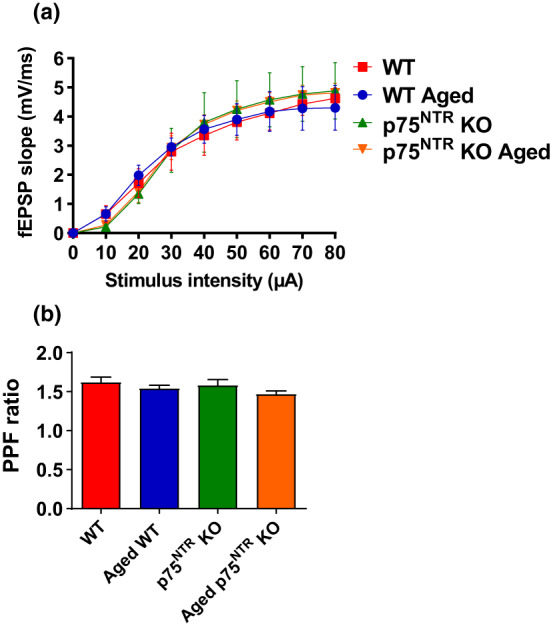
Aging does not affect basal transmission and neurotransmitter release in hippocampus. (a) Basal synaptic transmission was assayed by measuring fEPSPs within the stratum radiatum of the CA1 region of the hippocampus evoked by a bipolar electrode placed at the CA3‐CA1 border. No significant difference was observed in basal transmission among WT and p75^NTR^ KO mice in both age groups. (b) Paired‐pulse facilitation at the Schaffer collateral CA1 synapse was measured by dividing the amplitude of the second fEPSP by the amplitude of the first elicited by a pair of two 50‐ms spaced stimuli. No significant change was observed in PPF ratio in both age groups between WT and p75^NTR^ KO mice

To elucidate whether p75^NTR^ plays a role in synaptic plasticity changes associated with aging, we compared late‐LTP between young adult and aged p75^NTR^ KO mice. We found that late‐LTP in young adult p75^NTR^ KO mice was indistinguishable from that observed in young WT mice (red circles; Figure 2d). The mean fEPSP value immediately after the first tetanization, 142.02 ± 12.63%, was statistically different from the baseline (Wilcox test, *p* = 0.03), and the potentiation was maintained for 4 h (159.82 ± 9.02%, Wilcox test, *p* = 0.02; *U* test, *p* = 0.001; Figure 2d). To test whether aging impairs late‐LTP in p75^NTR^ KO mice, we applied STET to induce late‐LTP at synaptic input S1 in aged p75^NTR^ KO mice. The fEPSP slope value after the first tetanization was 169.62 ± 9.90% (Wilcox test, *p* = 0.008), and the potentiation was maintained throughout the entire recording period of 4 h (169.39 ± 10.60%, Wilcox test, *p* = 0.008; *U* test, *p* = 0.0002; Figure 2e). Four hours after LTP, the magnitude of potentiation was not significantly changed in aged p75^NTR^ KO mice compared to young adult p75^NTR^ KO mice (Figure 2f). We studied LTP decay by comparing 120‐, 180‐, and 240‐min LTP potentiation point to 30‐min LTP potentiation point. We observed that there was no significant LTP decay in aged WT mice compared other three groups (Figure 2g). To test the activation of NMDA receptor during the induction of late‐LTP in aged hippocampal neurons, the receptor antagonist AP5 (50 μM) was bath applied for 30 min before and after the induction of late‐LTP by STET in S1 (Figure 3a,b) in both aged WT and p75^NTR^ KO mice. No potentiation was observed in S1 (red circles), and both S1 in aged WT (103.19 ± 4.32%, Wilcox test, *p* = 0.843; *U* test, *p* = 0.679; Figure 3a) and aged p75^NTR^ KO mice (95.46 ± 1.49%, Wilcox test, *p* = 0.312; *U* test, *p* = 0.240; Figure 3b) remained at baseline level throughout the entire recording period of 4 h. The baseline in synaptic input S2 (blue circles) was stable throughout the recording period in all experiments. Furthermore, our Western blot analysis showed that aging significantly decreased the levels of hippocampal NR2A and NR2B in WT mice (Figure 3c,d), consistent with earlier findings (Magnusson, 2000; Zhao et al., 2009). Intriguingly, the age‐dependent decrease in hippocampal NR2A and NR2B levels was not observed in p75^NTR^ KO mice, which showed comparable levels in both young and old hippocampi (Figure 3c,d). These findings show that deletion of p75^NTR^ prevents the deficits in late‐LTP caused by aging, suggesting an essential role of p75^NTR^ in mediating age‐associated changes on hippocampal neuronal plasticity. In addition, NMDA receptor activity was essential for the reinstatement of late‐LTP in aged p75^NTR^ KO mice. We also assessed input–output relationship by plotting stimulation intensity and fEPSP slope. No significant difference was observed between young and old groups (Figure 4a), in consistent with a previous study (Yang et al., 2019). Additionally, there was no significant difference in paired‐pulse facilitation between young and old from both WT and p75^NTR^ KO mice (Figure 4b), in consistent with an earlier study (Arias‐Cavieres et al., 2017). These data suggest that aging does not affect basal synaptic transmission and probability of neurotransmitter release.

### 2.2 Deletion of p75^NTR^ prevents age‐associated NMDAR‐ and mGluR‐LTD enhancement

**FIGURE 5:**
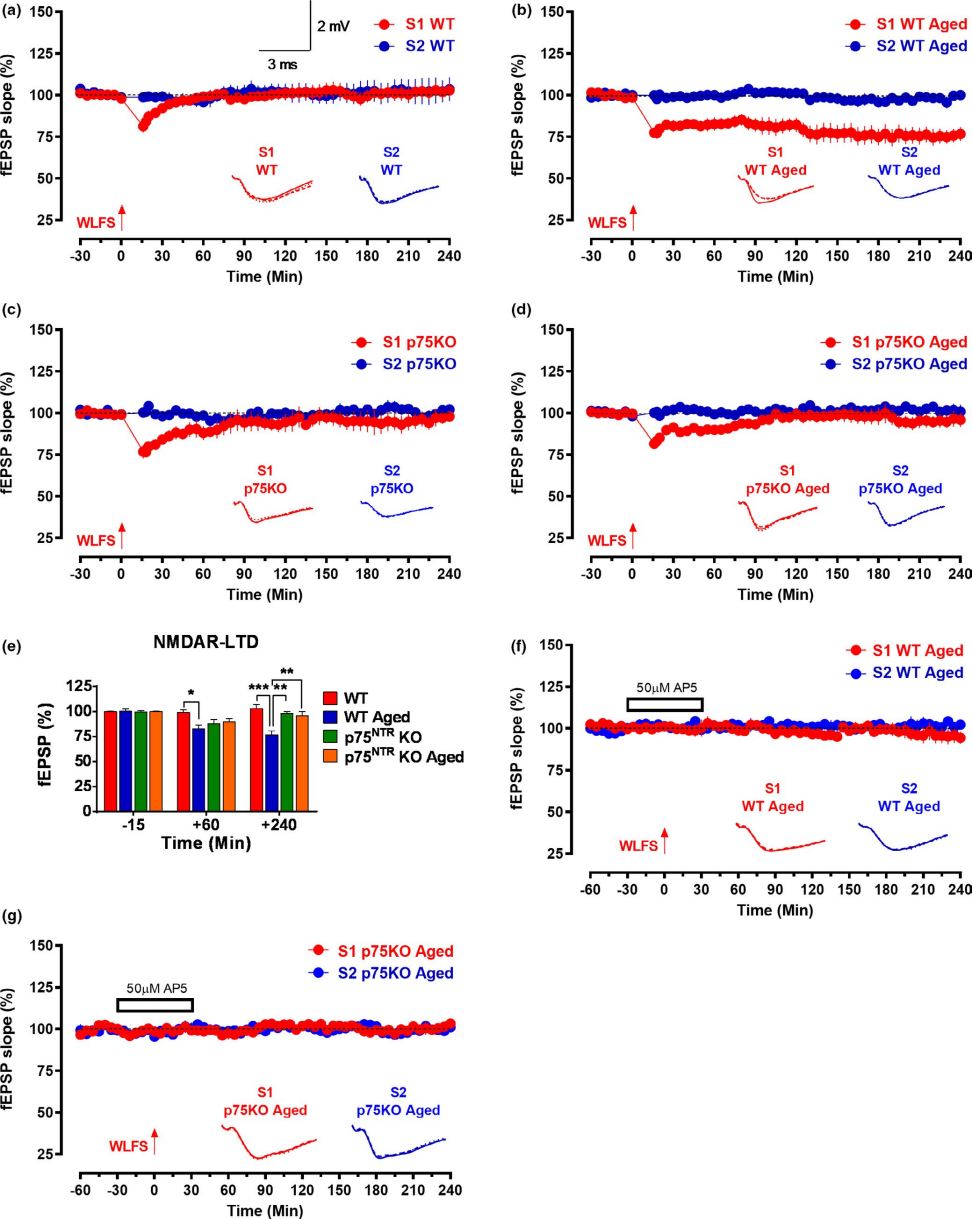
p75^NTR^ KO displayed normal NMDAR‐LTD irrespective of age. (a) The WLFS in S1 (red circles) resulted in early‐LTD which gradually returned to baseline in young adult WT mice (*N* = 7). (b) The WLFS in S1 (red circles) resulted in late‐LTD that was significantly maintained toward the end of the recording in aged WT mice (*N* = 6). The WLFS in S1 (red circles) resulted in early‐LTD, which gradually returned to baseline in (c) young adult p75^NTR^ KO mice (*N* = 6) and (d) aged p75^NTR^ KO mice (*N* = 7). (e) A histogram of mean fEPSP slope values recorded for young adult WT, aged WT, young adult p75^NTR^ KO, and aged p75^NTR^ KO mice at three different time points: 15 min (baseline), +60 min, and +240 min after LTD. At +60 min, the mean fEPSP slope value was significantly smaller in aged WT mice (*p* = 0.0111) compared to young adult WT. At +240 min, the mean fEPSP slope value was significantly smaller in aged WT mice compared to young adult WT (*p* = 0.0002), young adult p75^NTR^ KO (*p* = 0.0026), and aged p75^NTR^ KO mice (*p* = 0.0017). Asterisk indicates significant differences between groups (two‐way ANOVA, *p < 0.05, **p < 0.01, ***p < 0.001). Error bars indicate ±SEM. The WLFS in S1 (red circles) could not induce depression in the presence of AP5 (50 μM) in (f) aged WT (*N* = 6) and (g) aged p75^NTR^ KO (*N* = 7) mice. Control potentials from S2 (blue circles) remained stable during the recording period in all experiments. Analog traces represent typical fEPSPs of inputs S1 and S2 15 min before (solid line), 60 min after (dashed line) tetanization, and at the end of the recording (dotted line). One solid arrow represents WLFS for the induction of early‐LTD. Scale bars for all the traces vertical: 2 mV; horizontal: 3 ms. Error bars indicate ± SEM

LTD dependent on the N‐methyl‐D‐aspartate receptor (NMDAR‐LTD) has previously been reported to increase with age (Norris et al., 1996). We performed experiments to discern the effects of aging on early‐LTD in Schaffer collateral‐CA1 synapses of hippocampal slices derived from either young adult or aged WT and p75^NTR^ KO mice. A single‐weak, low‐frequency stimulation (WLFS, 1 Hz, 900 pulses) resulted in early‐LTD (red circles), which gradually returned to baseline in young adult WT mice (Figure 5a). The first mean fEPSP slope value after WLFS was 81.32 ± 3.74% (Wilcox test, *p* = 0.02), and the depression in S1 was maintained for up to 35 min (Wilcox test, *p* = 0.047) or 25 min (*U* test, *p* = 0.026; Figure 5a). In aged WT mice, the first mean fEPSP slope value after WLFS was comparable to young mice (77.47 ± 2.62%; Wilcox test, *p* = 0.03). However, the depression in S1 was maintained toward the end of the recording (76.66 ± 4.16%, Wilcox test, *p* = 0.03; *U* test, *p* = 0.002; Figure 5b), in contrast to WT mice, in which S1 depression was only transient. Four hours after LTD induction, the mean fEPSP was significantly smaller (*p* = 0.0002) in aged WT mice compared to young adult WT mice (Figure 5e). The baseline in synaptic input S2 (blue circles) for both young adult and aged WT mice was stable throughout the recording period in all experiments (Figure 5a,b). These data show that aging enhances NMDAR‐LTD by transforming a short‐lasting form of LTD to a long‐lasting one.

We then compared the development of early‐LTD between young adult and aged p75^NTR^ KO mice. Similar to WT mice, early‐LTD induction in hippocampal slices derived from both young adult and aged p75^NTR^ KO mice resulted in a decaying form of LTD (Figure 5c,d). The first fEPSP slope value after WLFS in young adult was 76.87 ± 4.07% (Wilcox test, *p* = 0.03; Figure 5c) and for aged mice was 81.63 ± 2.48% (Wilcox test, *p* = 0.016; Figure 5d), both values very similar to those obtained in WT mice. Also similar to WT mice, the depression in fEPSP in S1 was maintained up to 30 min (Wilcox test, *p* = 0.03) or up to 45 min (*U* test, *p* = 0.015) after WLFS in young adult p75^NTR^ KO mice (Figure 5c). In contrast to WT mice, however, S1 depression was maintained for only to 90 min (Wilcox test, *p* = 0.026) or 85 min (*U* test, *p* = 0.026) for aged p75^NTR^ KO mice and was statistically indistinguishable from baseline at the end of the recording (Figure 5d). To test the activation of NMDA receptor during the induction of LTD in aged hippocampal neurons, the receptor antagonist AP5 (50 μM) was bath applied for 30 min before and after the induction of LTD by WLFS in S1 (Figure 5f,g) in both aged WT and p75^NTR^ KO mice. No depression was observed in S1 (red circles), and both S1 in aged WT (94.41 ± 3.56%, Wilcox test, *p* = 0.156; *U* test, *p* = 0.132; Figure 5f) and aged p75^NTR^ KO (103.30 ± 2.58%, Wilcox test, *p* = 0.218; *U* test, *p* = 0.393; Figure 5g) mice remained at baseline level throughout the entire recording period of 4 h. The baseline in synaptic input S2 (blue circles) was stable throughout the recording period in all experiments. These results show that deletion of p75^NTR^ prevents age‐associated changes in NMDAR‐LTD.

**FIGURE 6:**
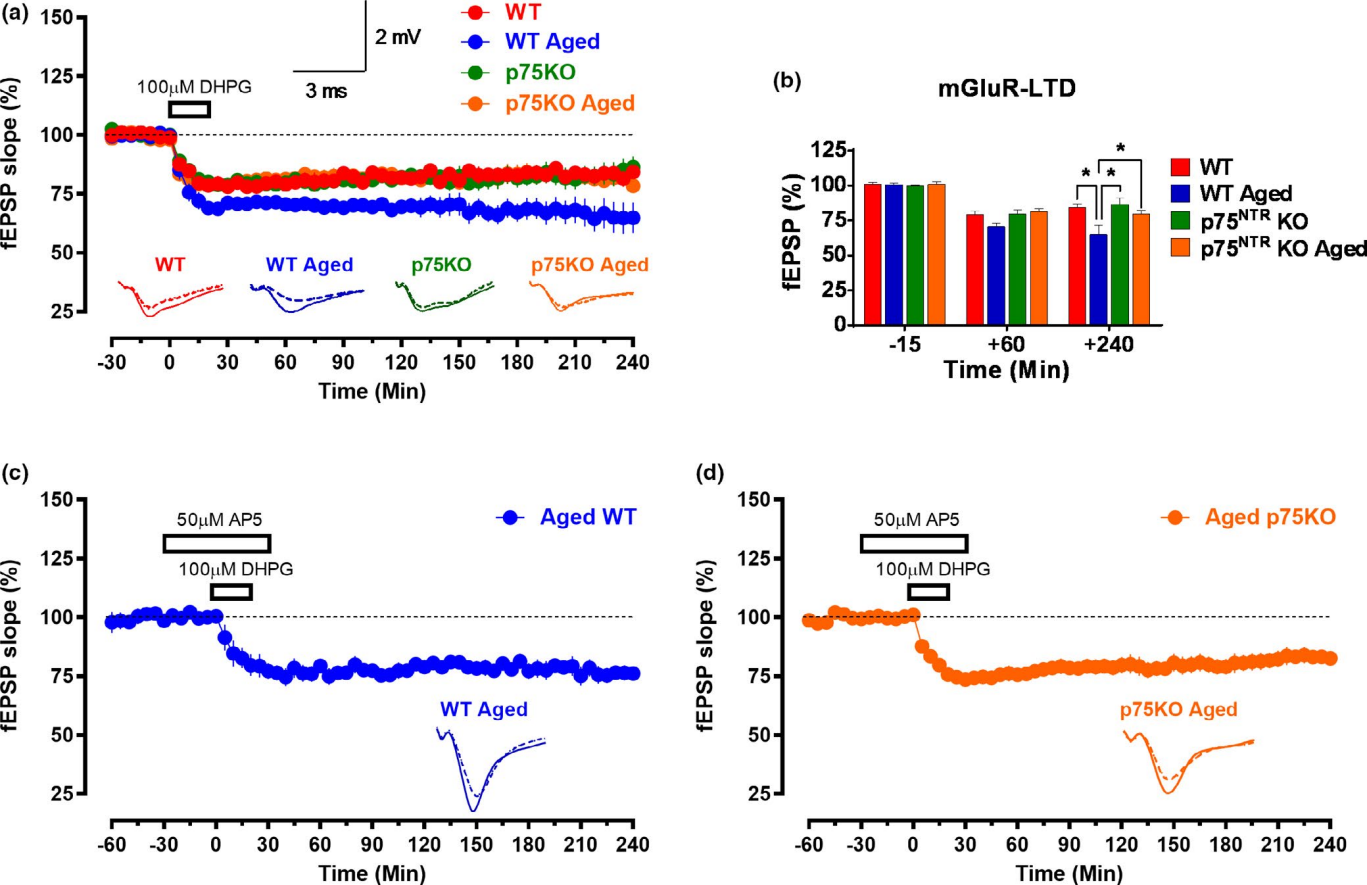
p75^NTR^ KO displayed normal mGluR‐LTD irrespective of age. (a) Bath application of 100 µM DHPG for 20 min resulted in mGluR‐LTD that persisted till the end of the recording in young adult WT, aged WT, young adult p75^NTR^ KO, and aged p75^NTR^ KO mice. (b) A histogram of mean fEPSP slope values recorded for young adult WT, aged WT, young adult p75^NTR^ KO, and aged p75^NTR^ KO mice at three different time points: 15 min (baseline), +60 min, and +240 min after LTD. At +240 min, the mean fEPSP slope value was significantly smaller in aged WT mice compared to young adult WT (*p* = 0.0120), young adult p75^NTR^ KO (*p* = 0.0100), and aged p75^NTR^ KO mice (*p* = 0.0115). Asterisk indicates significant differences between groups (two‐way ANOVA, *p < 0.05). Error bars indicate ± SEM. Bath application of 100 µM DHPG for 20 min in the presence of AP5 (50 μM) resulted in mGluR‐LTD that persisted till the end of the recording in (c) aged WT (*N* = 6) and (d) aged p75^NTR^ KO (*N* = 7) mice. Analog traces represent typical fEPSPs of 15 min before (solid line), 60 min after (dashed line) tetanization, and at the end of the recording (dotted line). Scale bars for all the traces vertical: 2 mV; horizontal: 3 ms. Error bars indicate ± SEM

Aging has also been known to affect metabotropic glutamate receptor (mGluR)‐LTD (Kumar & Foster, 2007, 2014), which can be induced by bath application of the group I selective agonist DHPG (Fitzjohn et al., 1999; Gladding et al., 2009; Kumar & Foster, 2007, 2014; Palmer et al., 1997; Sharma & Sajikumar, 2018). To investigate mGluR‐LTD, single‐pathway experiments were performed in all the electrophysiological recordings. In young adult WT mice, bath application of 100 µM DHPG for 20 min resulted in mGluR‐LTD that persisted till the end of the recording (Figure 6a). The observed LTD was statistically different from baseline from the 5^th^ min onward (87.55 ± 2.14%, Wilcox test, *p* = 0.015) and lasted till the end of the recording at four hours (84.24 ± 2.55%, Wilcox test, *p* = 0.015; Figure 6a). In aged WT mice, the observed DHPG induced mGluR‐LTD was also significant from the 5^th^ min (85.34 ± 4.41%, Wilcox test, *p* = 0.015) till the end of the recording (64.80 ± 6.53, Wilcox test, *p* = 0.015; Figure 6a). In contrast to young mice, however, the mean fEPSP at 240 min was significantly smaller (*p* = 0.0120) in aged WT mice (Figure 6b). Bath application of DHPG also led to mGluR‐LTD that maintained toward the end of the recording (86.41 ± 4.58%, Wilcox test, *p* = 0.04) in young adult p75^NTR^ KO mice (Figure 6a). In aged p75^NTR^ KO mice, the DHPG‐induced mGluR‐LTD also persisted till the end of the recording (78.38 ± 2.36%, Wilcox test, *p* = 0.03; Figure 6a). However, and in contrast to aged WT mice, the mean fEPSP at 240 min in the aged p75^NTR^ KO mice was not significantly different from that observed in young mice, both WT and p75^NTR^ KO (Figure 6b). To test whether activation of NMDA receptor is required for the induction of mGluR‐LTD in aged hippocampal neurons, the receptor antagonist AP5 (50 μM) was bath applied for 30 min before and after the induction of mGluR‐LTD by DHPG in S1 (Figure 6c,d) in both aged WT and p75^NTR^ KO mice. Depression was observed in S1 (red circles) and S1 in both aged WT (76.14 ± 3.02%, Wilcox test, *p* = 0.031; Figure 6c) and aged p75^NTR^ KO (82.59 ± 3.04%, Wilcox test, *p* = 0.015; Figure 6d) mice, which were maintained throughout the entire recording period of 4 h. These data show that aging increases mGluR‐LTD and that such increase can be prevented by the deletion of p75^NTR^. However, enhancement of mGluR‐LTD in aged WT mice was not dependent on NMDA receptor.

### 2.3 Deletion of p75^NTR^ prevents age‐associated STC deficits

**FIGURE 7:**
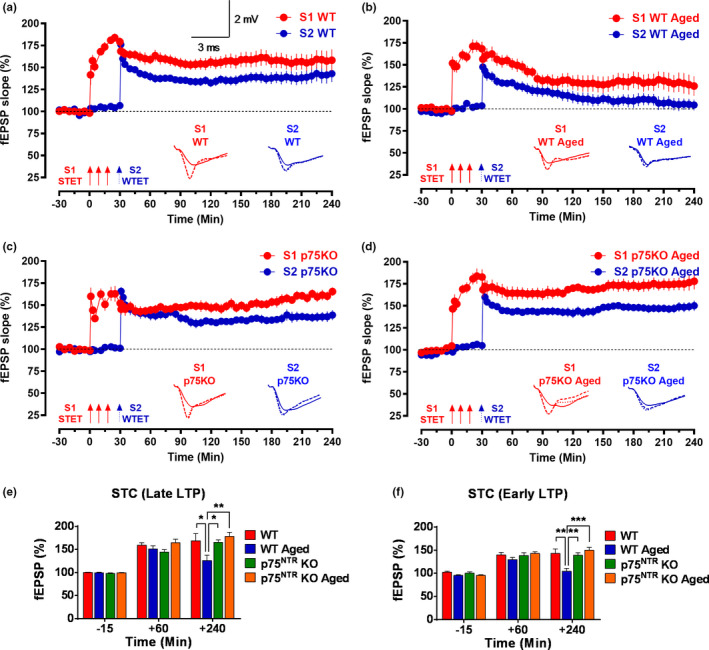
p75^NTR^ KO displays normal synaptic tagging and capture (STC) irrespective of age. “Strong before weak” paradigm was used to study STC. (a) Both STET in S1 (red circles) and WTET in S2 (blue circles) resulted in a significant potentiation that maintained till the end of recording in young adult WT mice (*N* = 7). (b) Both STET in S1 (red circles) and WTET in S2 (blue circles) resulted in early‐LTP that was not reinforced into late‐LTP in aged WT mice (*N* = 7). (c) Both STET in S1 (red circles) and WTET in S2 (blue circles) resulted in a significant potentiation that maintained till the end of experiment in young adult p75^NTR^ KO mice (*N* = 8). (d) Both STET in S1 (red circles) and WTET in S2 (blue circles) resulted in a significant potentiation that maintained till the end of recording in aged p75^NTR^ KO mice (*N* = 8). Control potentials from S2 (blue circles) remained stable during the recording period in all experiments. Scale bars for all the traces vertical: 2 mV; horizontal: 3 ms. Error bars indicate ± SEM. Symbols and analog traces as in Figure 1. (e) A histogram of mean fEPSP slope values recorded for young adult WT, aged WT, young adult p75^NTR^ KO, and aged p75^NTR^ KO mice at three different time points: 15 min (baseline), +60 min, and +240 min after late‐LTP for STC. At +240 min, the mean fEPSP slope value was significantly smaller in aged WT mice compared to young adult WT (*p* = 0.0489), young adult p75^NTR^ KO (*p* = 0.0143) and aged p75^NTR^ KO mice (*p* = 0.0020). (f) A histogram of mean fEPSP slope values recorded for young adult WT, aged WT, young adult p75^NTR^ KO and aged p75^NTR^ KO mice at three different time points: 15 min (baseline), +60 min, and +240 min after early‐LTP for STC. At +240 min, the mean fEPSP slope value was significantly smaller in aged WT mice compared to young adult WT (*p* = 0.0016), young adult p75^NTR^ KO (*p* = 0.0023), and aged p75^NTR^ KO mice (*p* = 0.0001). Asterisk indicates significant differences between groups (two‐way ANOVA, *p < 0.05, **p < 0.01, ***p < 0.001). Error bars indicate ± SEM

Having established the critical effects of p75^NTR^ on LTP during aging, we set out to assess whether p75^NTR^ also impacts associative plasticity in aged mice using the STC protocol. To investigate STC, we used the “strong before weak” (SBW) experimental paradigm, in which late‐LTP is induced in S1 by STET 30 min before the induction of early‐LTP by weak tetanization (WTET) in S2 (Figure 7a–d). First, we studied STC in hippocampal slices from young adult WT mice. The post‐tetanization potential in S1 was significantly elevated (141.55 ± 11.53%, Wilcox test, *p* = 0.04) immediately after tetanization and maintained at high levels for up to 4 h (158.15 ± 12.45%, Wilcox test, *p* = 0.02; Figure 7a). In S2, WTET led to a statistically significant potentiation (176.08 ± 5.50%, Wilcox test, *p* = 0.02) immediately after tetanization and stayed up to the end of the recording (142.98 ± 9.64%, Wilcox test, *p* = 0.02; Figure 7a). These results confirm the expression of STC in young adult WT mice. In contrast, the same experimental procedure failed to elicit STC in hippocampal slices of aged WT mice (Figure 7b). Although post‐tetanization potentiation in S1 was achieved immediately after STET (151.86 ± 7.79%, Wilcox test, *p* = 0.008), potentiation remained for only 3 h (Wilcox test, *p* = 0.04; Figure 7b). Four hours after induction, the mean fEPSP was significantly lower (*p* = 0.0489) in aged WT mice compared to young adult WT mice (Figure 7e). In aged WT mice, the post‐tetanization potentials in S2 were significantly elevated (147.58 ± 7.86%, Wilcox test, *p* = 0.02) after the WTET but only until 125 min (Wilcox test, *p* = 0.04), after which the potentiation decayed rapidly, reaching baseline values at the end of the recording (Figure 7b). The mean fEPSP at 4 h was significantly smaller (*p* = 0.0016) in aged WT mice compared to young adult mice (Figure 7f). Together, these results indicated that aging disrupts the expression of STC in hippocampal neurons.

Next, we investigated the expression of STC in p75^NTR^ KO mice. In young adult animals, induction of late‐LTP in S1 by STET led to significant potentiation (159.99 ± 10.07%, Wilcox test, *p* = 0.008) immediately after tetanization which remained stable for up to 4 h (Wilcox test, *p* = 0.008; Figure 7c). Similarly, induction of early‐LTP by WTET also resulted in significant potentiation (165.59 ± 4.77%, Wilcox test, *p* = 0.008) in S2 after the first tetanization, and this also remained stable till the end of the recording (Wilcox test, *p* = 0.008; Figure 7c). Thus, STC induction in young p75^NTR^ KO mice was indistinguishable from that in WT mice. Interestingly, and in contrast to WT mice, aged p75^NTR^ KO mice displayed normal STC that was comparable to that observed in young adult animals, either WT or KO (Figure 7d). Significant potentiation (146.58 ± 7.58%, Wilcox test, *p* = 0.008) was observed in S1 after STET which remained stable till the end of the recording (Wilcox test, *p* = 0.008; Figure 7d). In S2, WTET also yielded significant potentiation (168.16 ± 7.53%, Wilcox test, *p* = 0.008) immediately after induction, which was maintained until the end of the recording (Wilcox test, *p* = 0.008; Figure 7d). These results show that deletion of p75^NTR^ prevents the deficits in hippocampal associative plasticity caused by aging.

### 2.4 Deletion of p75^NTR^ restores age‐associated deficits in associative memory

**FIGURE 8:**
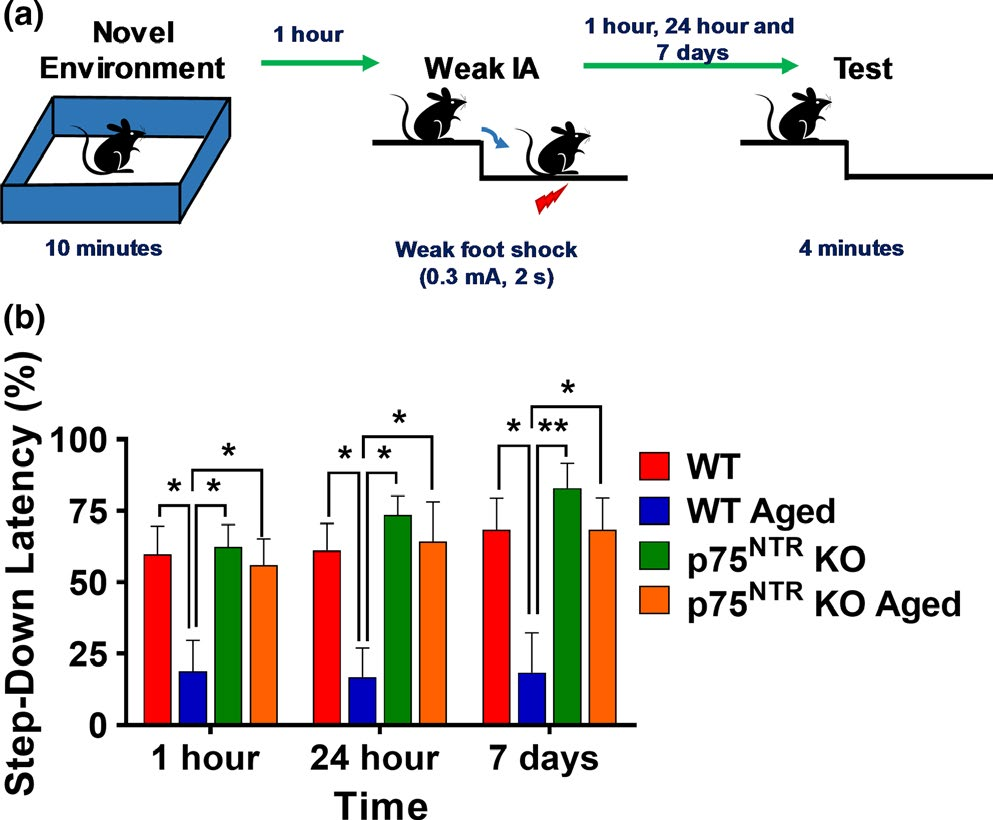
p75^NTR^ KO displays normal associative memory irrespective of age. (a) Schematic diagram of behavioral tagging experiment protocol. A mouse was being placed in a novel environment/open field (OF) for 10 min within 1 hour before weak inhibitory avoidance (IA). Step‐down latency was tested at 1 h, 24 h, and 7 d post‐IA. (b) Associative memory was impaired in aged WT mice. Associative memory was, however, normal in aged p75^NTR^ KO mice. Asterisks indicate significant differences between groups (two‐way ANOVA, *p < 0.05, **p < 0.01). Error bars indicate ± SEM

We investigated whether the effects of aging and p75^NTR^ KO on synaptic plasticity were reflected in behavior by testing associative memory. To this end, we used a behavioral tagging paradigm, in which mice are exposed to an open field (OF) that serves as a novel environment before application of a weak foot shock (Figure 8a). The strength of associative memory was measured as the latency to step down to bars that were associated with a weak foot shock at various time points (1 h, 24 h, and 7 days). A longer step‐down latency indicates a stronger associative memory as the mice would stay on the platform for a longer time before stepping down to the bars. Aged WT mice that experienced a foot shock after OF exploration had a significantly lower step‐down latency compared with the young adult WT mice at all three different time points (*p* = 0.0336 for 1 h, *p* = 0.0369 for 24 h, and *p* = 0.0380 for 7 days; Figure 8b). This suggested an impairment in memory acquisition and/or retention in aged WT mice. In contrast, both young adult and aged p75^NTR^ KO mice exhibited normal memory that was maintained for up to 7 days (Figure 8b). Strikingly, the performance of aged p75^NTR^ KO mice was essentially indistinguishable from that of young mice. These behavioral tagging experiments show that associative memory of aged p75^NTR^ KO remained intact and comparable to that of young control mice, further supporting the important role of p75^NTR^ in the plasticity and memory impairments that occur as a consequence of normal aging.

### 2.5 Deletion of p75^NTR^ prevents age‐mediated changes in BDNF and apoptosis pathways

**FIGURE 9:**
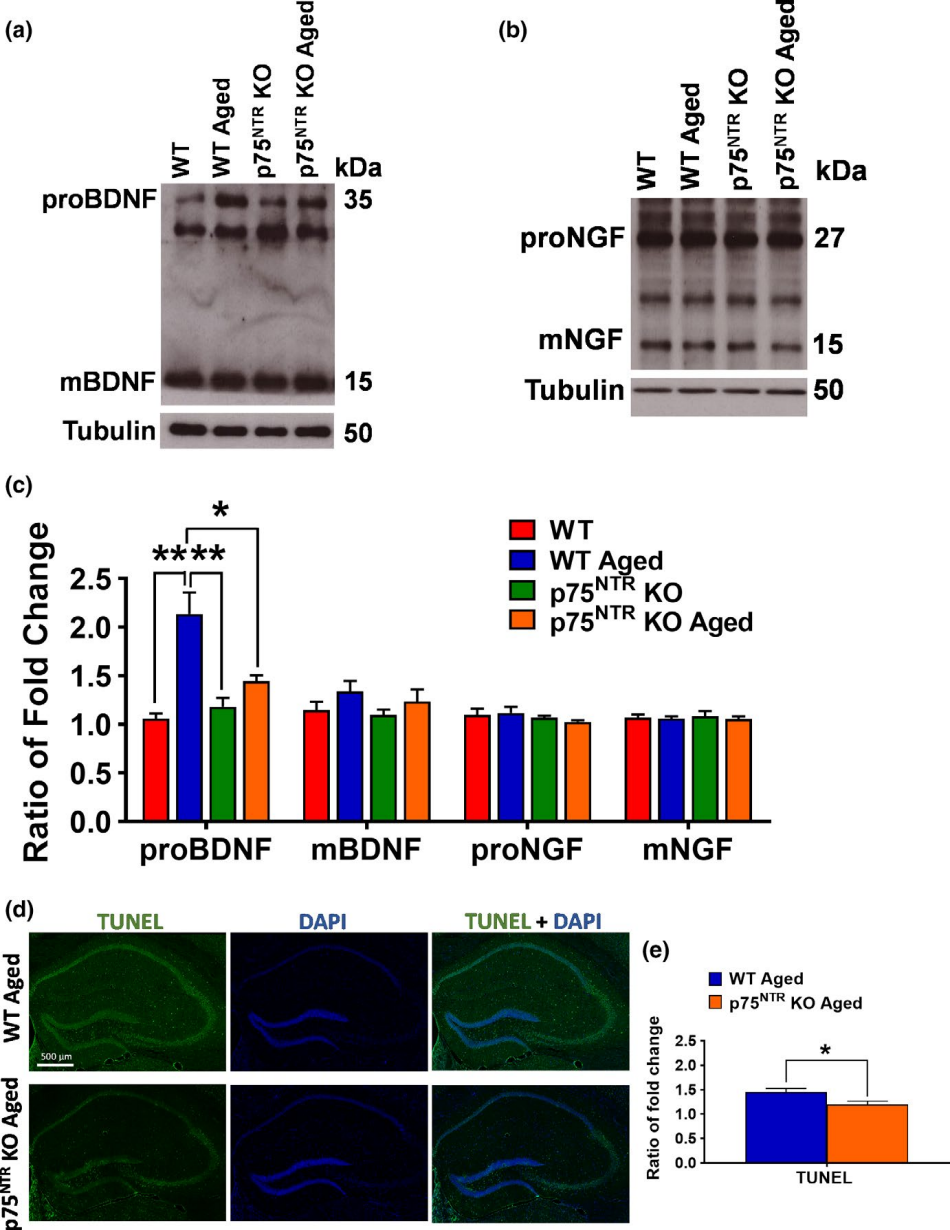
Effects of age on BDNF and NGF levels, and apoptosis in the hippocampus. (a) Western blot analysis of hippocampal BDNF levels between young adult and aged from WT and p75^NTR^ KO mice. (b) Western blot analysis of hippocampal NGF levels between young adult and aged from WT and p75^NTR^ KO mice. (c) Ratio of fold change from Western blot. The proBDNF level was significantly increased in aged WT mice compared to young adult WT (*p* = 0.0060), young adult p75^NTR^ KO (*p* = 0.0052), and aged p75^NTR^ KO mice (*p* = 0.0111), *N* = 4 for each group. No significant difference in mature BDNF level from aged WT mice compared to young adult WT, young adult p75^NTR^ KO, and aged p75^NTR^ KO mice, *N* = 4, for each group. No significant change in the levels of NGF from aged WT mice compared to young adult WT, young adult p75^NTR^ KO, and aged p75^NTR^ KO mice, *N* = 3 for each group. The values of the individual groups were calculated in relation to the control group, while tubulin serves as a loading control. Asterisk indicates significant differences between groups (two‐way ANOVA, *p < 0.05, **p < 0.01). Error bars indicate ± SEM. (d) Representative photomicrographs of TUNEL staining (green) and counterstained with DAPI (dark blue) immunohistochemistry in hippocampus of aged WT and p75^NTR^ KO mice. (e) Quantitation of relative immunofluorescence intensity of TUNEL signal was shown as a bar graph. The fold change of TUNEL fluorescence in aged WT mice was significantly higher (*p* = 0.0336) than aged p75^NTR^ KO mice, *N* = 3 for each group. Asterisk indicates significant differences between groups (unpaired *t*‐test, *p < 0.05). Error bars indicate ± SEM

Next, we investigated the possible mechanisms by which deletion of p75^NTR^ prevents age‐mediated deficits in synaptic plasticity. Through its action on p75^NTR^, proBDNF has been shown to exert detrimental effects on synaptic plasticity and memory (Kailainathan et al., 2016; Yang et al., 2014). Importantly, proBDNF levels in the hippocampus have been shown to increase with aging in mice (Buhusi et al., 2017). We confirmed that aging significantly increased (*p* = 0.0060) the levels of hippocampal proBDNF in WT mice (Figure 9a,c). Intriguingly, the age‐dependent increase in hippocampal proBDNF levels was not observed in p75^NTR^ KO mice, which showed comparable levels in both young and old hippocampi (Figure 9a,c). We did not detect a significant difference in the levels of hippocampal mature BDNF between young adult and aged mice, either WT or p75^NTR^ KO (Figure 9a,c). In addition, no significant change in the level of hippocampal NGF between young adult and aged from WT and p75^NTR^ KO mice was observed (Figure 9b,c). Previous studies have shown that proNGF was significantly increased in aged rats (Perovic et al., 2013; Terry Jr et al., 2011). However, other studies have found no change in NGF level in the aged animals (Bimonte‐Nelson et al., 2007; Crutcher & Weingartner, 1991). ProBDNF is a potent inducer of neuronal death (Lee et al., 2001; Nykjaer & Willnow, 2012). Expression of proBDNF has been reported to be elevated in a number of neurodegenerative disorders and after neural injury (Iulita & Cuello, 2014; Volosin et al., 2008). Our results show that apoptotic cells in hippocampus as assessed by TUNEL staining were significantly higher (*p* = 0.0336) in aged WT mice compared to aged p75^NTR^ KO mice (Figure 9d–e). Altogether, these data demonstrate that p75^NTR^ together with proBDNF, contributing to neuronal death in aging.

### 2.6 Deletion of p75^NTR^ prevents age‐mediated changes in MAPK pathway

**FIGURE 10:**
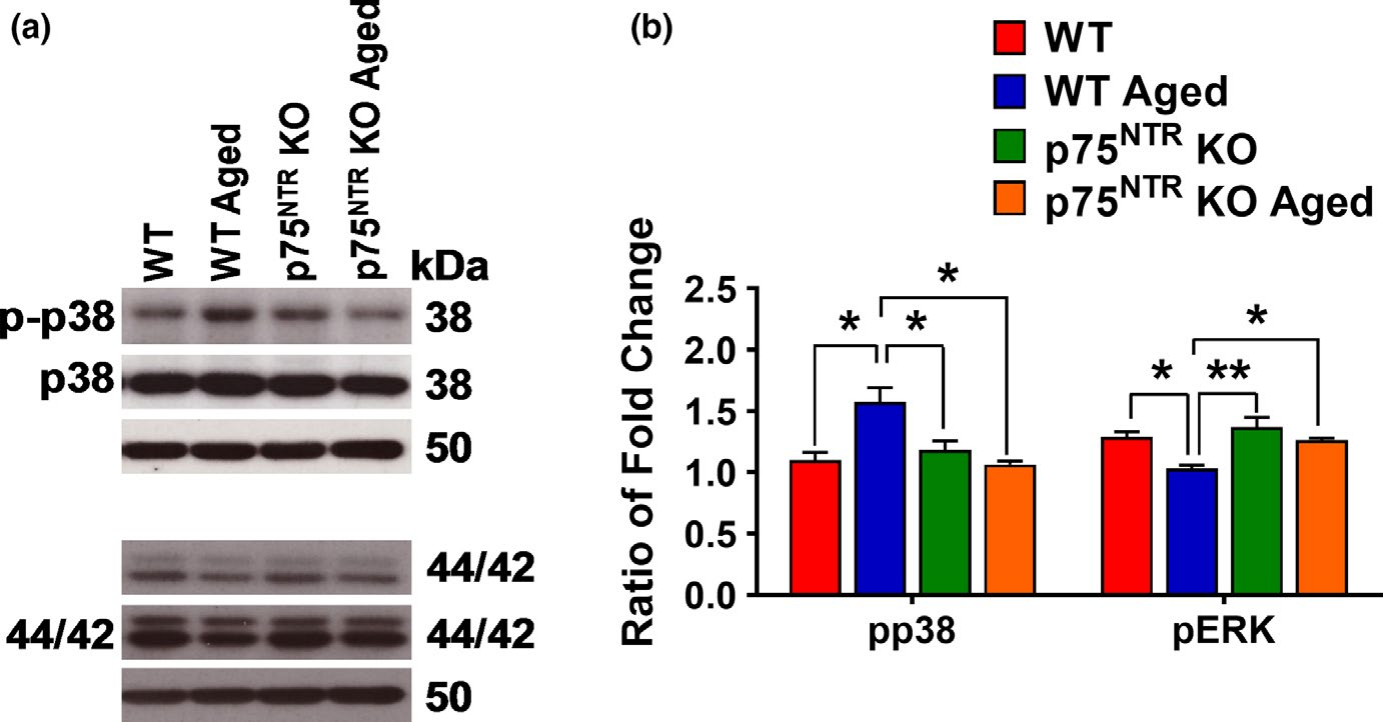
Effects of age on p38 and ERK1/2 levels in the hippocampus. (a) Western blot analysis of hippocampal p38 and ERK1/2 levels between young adult and aged from WT and p75^NTR^ KO mice. (b) Ratio of fold change from Western blot. The phospho/total p38 ratio was significantly increased in aged WT mice compared to young adult WT (*p* = 0.0221), young adult p75^NTR^ KO (*p* = 0.0462), and age p75^NTR^ KO (*p* = 0.0162) mice, *N* = 3 for each group. The phospho/total ERK1/2 ratio was significantly decreased in aged WT mice compared to young adult WT (*p* = 0.0299), young adult p75^NTR^ KO (*p* = 0.0088), and aged p75^NTR^ KO (*p* = 0.0447) mice, *N* = 3 for each group. The values of the individual groups were calculated in relation to the control group, while tubulin serves as a loading control. Asterisk indicates significant differences between groups (two‐way ANOVA, *p < 0.05, **p < 0.01). Error bars indicate ± SEM

The mitogen‐activated protein kinase (MAPK) pathway, which among others involves p38 and extracellular signal‐regulated kinases (ERK1/2), plays a critical in the regulation of synaptic plasticity (Bolshakov et al., 2000). We found that phosphorylation of p38 was significantly increased (*p* = 0.0221) in aged WT mice, but not p75^NTR^ KO (Figure 10a,b). In contrast, phosphorylation of ERK1/2 was significantly decreased (*p* = 0.0299) in aged WT but not in aged p75^NTR^ KO (Figure 10a,b). Taken together, these results show that aging increases p38 activation, while decreases ERK activation in the mouse hippocampus.

### 2.7 Deletion of p75^NTR^ prevents age‐mediated changes in RhoA‐ROCK2‐LIMK1‐cofilin and Arc pathways

**FIGURE 11:**
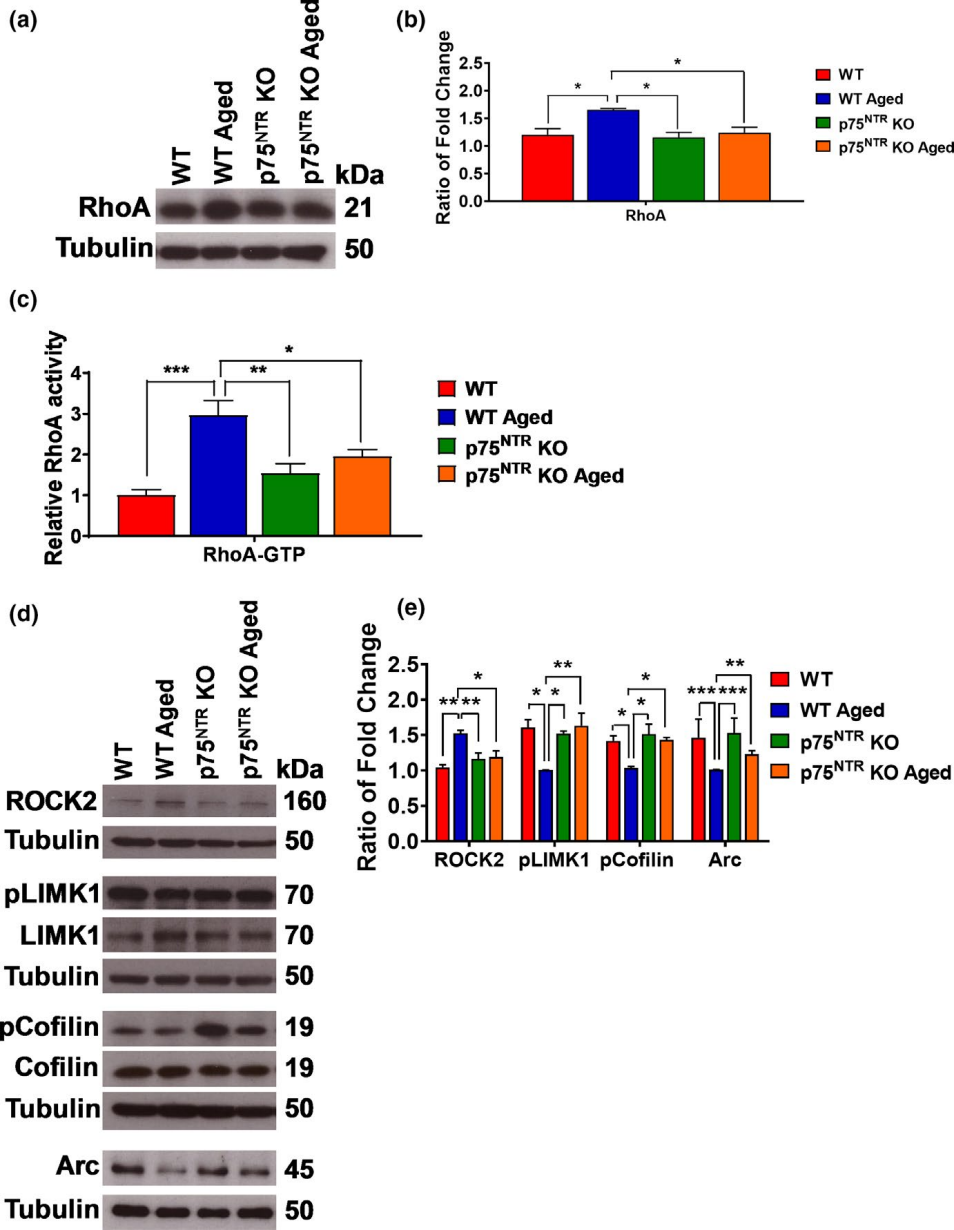
Effects of age on RhoA, ROCK2, LIMK1, cofilin, and Arc levels in the hippocampus. (a) Western blot analysis of hippocampal RhoA level between young adult and aged from both WT and p75^NTR^ KO mice. (b) Ratio of fold change from Western blot. The RhoA level was significantly increased in aged WT mice compared to young adult WT (*p* = 0.0338), young adult p75^NTR^ KO (*p* = 0.0206), and aged p75^NTR^ KO (*p* = 0.0488) mice, *N* = 4 for each group. (c) ELISA analysis of hippocampal RhoA‐GTP activity between young adult and aged from both WT and p75^NTR^ KO mice. The RhoA‐GTP activity was significantly increased in aged WT mice compared to young adult WT (*p* = 0.0002), young adult p75^NTR^ KO (*p* = 0.0021), and aged p75^NTR^ KO (*p* = 0.0192) mice, *N* = 5 for each group. (d) Western blot analysis of hippocampal ROCK2, LIMK1, cofilin, and Arc levels between young adult and aged from both WT and p75^NTR^ KO mice. (e) Ratio of fold change from Western blot. The ROCK2 level was significantly increased in aged WT mice compared to young adult WT (*p* = 0.0012), young adult p75^NTR^ KO (*p* = 0.0085), and aged p75^NTR^ KO (*p* = 0.0135) mice, *N* = 4 for each group. The phosphorylated LIMK1 protein level was significantly decreased in aged WT mice compared to young adult WT (*p* = 0.0111), young adult p75^NTR^ KO (*p* = 0.0223), and aged p75^NTR^ KO (*p* = 0.0091) mice, *N* = 3 for each group. The phosphorylated cofilin level was significantly decreased in aged WT mice compared to young adult WT (*p* = 0.0499), young adult p75^NTR^ KO (*p* = 0.0201), and aged p75^NTR^ KO (*p* = 0.0427) mice, *N* = 3 for each group. The Arc level was significantly decreased in aged WT mice compared to young adult WT (*p* = 0.0008), young adult p75^NTR^ KO (*p* = 0.0002), and aged p75^NTR^ KO (*p* = 0.0017) mice, *N* = 4 for each group. The values of the individual groups were calculated in relation to the control group, while tubulin serves as a loading control. Asterisk indicates significant differences between groups (two‐way ANOVA, *p < 0.05, **p < 0.01, ***p < 0.001). Error bars indicate ± SEM

RhoA‐ROCK‐LIMK‐cofilin pathway is a well‐known signaling cascade for the regulation of actin dynamics (Hsieh et al., 2006; Ohashi et al., 2000). It has been previously reported that p75^NTR^ signaling can regulate the activity of the Ras homolog gene family member A (RhoA) (Yamashita et al., 1999), binding and recruiting to the cell membrane the RhoA regulator RhoGDI (Yamashita et al., 1999). We found that aging significantly increased (*p* = 0.0338) RhoA protein levels in aged WT but not aged p75^NTR^ KO mice compared to young adult WT mice (Figure 11a,b), indicating a possible role for p75^NTR^ in the upregulation of RhoA protein during aging. In addition, RhoA activity was significantly increased in aged WT (*p* = 0.0002) but not aged p75^NTR^ KO mice compared to young adult WT mice (Figure 11c). The levels of the RhoA downstream target, Rho‐associated coiled‐coil containing protein kinase 2 (ROCK2), were also significantly increased (*p* = 0.0012) in WT but not p75^NTR^ KO during aging (Figure 11d,e). In addition, aging resulted in a significant decrease (*p* = 0.0111) in the phosphorylation of LIMK1 at threonine 508 in WT mice (Figure 11d,e). No significant change was observed in aged p75^NTR^ KO mice (Figure 11d,e). In turn, LIMK has been implicated in the regulation of cofilin activity (Endo et al., 2003). We found that cofilin phosphorylation at serine 3 was significantly lower (*p* = 0.0499) in aged WT mice compared with young adult WT mice (Figure 11d,e). In contrast, phosphorylated cofilin level was unchanged in aged p75^NTR^ KO mice (Figure 11d,e). Finally, we also investigated the levels of activity‐regulated cytoskeleton Arc/Arg3.1 protein, as this has been implicated in regulating spine density and, as a result, modulating plasticity mechanisms (Steward et al., 1998; Steward & Worley, 2001). We found that aging significantly decreased Arc/Arg3.1 protein levels in aged WT mice compared to young adult WT (*p* = 0.0008), young adult p75^NTR^ KO (*p* = 0.0002) and aged p75^NTR^ KO (*p* = 0.0017) mice (Figure 11d,e). Together, these data suggest that aging‐mediated upregulation of the RhoA‐ROCK2‐LIMK1‐cofilin and Arc pathways via p75^NTR^ may result in impaired actin dynamics, which is in turn critical for synaptic function and efficacy.

## 3 DISCUSSION

Activity‐dependent, bidirectional synaptic plasticity plays an important role in learning and memory, and is known to be affected during aging (Barnes et al., 1992; Deupree et al., 1993; Landfield et al., 1986). Our present study shows that aging influences the LTP and LTD balance in the hippocampus. Aged hippocampal CA3‐CA1 synapses exhibited a significant deficit in the magnitude of late‐LTP, although both the induction and persistence of LTP were not compromised, an observation consistent with our earlier reports (Sharma et al., 2015; Shetty et al., 2017). In contrast, our data show that both NMDAR‐ and mGluR‐LTD are augmented during aging. These results are consistent with previous studies showing that aged rodents are more susceptible to LTD (Norris et al., 1996) and the reversal of LTP (Foster & Norris, 1997) compared to young rodents. Additionally, the magnitude of mGluR‐LTD has been demonstrated to increase during aging (Kumar & Foster, 2014). Therefore, the present study together with earlier findings lend support to the notion that LTD is favored over LTP during aging, implicating a shift in the plasticity mechanisms that are associated with normal aging.

Synaptic associativity allows neural networks to encode stable long‐term memories by transforming short‐term plasticity into long‐term plasticity. Aging is known to impact associative plasticity and memory consolidation (Sharma et al., 2015; Shetty et al., 2017). Using the STC paradigm, our results demonstrate that aging disrupts associative plasticity. This is in line with our finding from behavioral tagging experiments, showing that aging impairs associative memory. It has been postulated that during behavioral tagging, PRPs synthesized under the influence of novelty, could transform transient forms of plasticity into long‐lasting forms (Moncada & Viola, 2007). Studies have demonstrated substantial behavioral changes in 12‐month‐old mice compared to young mice (Shoji & Miyakawa, 2019; Shoji et al., 2016), and our study showed that the expression of hippocampal p75^NTR^ was significantly higher in 12 months compared to 6‐week‐old mice. Thus, we postulated that an increase expression of p75^NTR^ might be at least in part responsible for behavioral changes in 12‐month‐old mice. Overall, the findings from our electrophysiological and behavioral tagging studies further support the negative impact of aging in hippocampal‐dependent long‐term memory and associative memory.

While there has been some evidence indicating a relationship between p75^NTR^ and cognitive decline, this has come mainly from studies of mouse models of neurodegenerative diseases (Bachis et al., 2016; Meeker & Williams, 2015). Our present study is the first attempt to investigate the effect of p75^NTR^ on cognitive decline associated with normal aging in the absence of overt disease. Our data show that p75^NTR^ KO mice exhibit normal late‐LTP, similar to previous studies (Rösch et al., 2005; Woo et al., 2005). Interestingly, deletion of p75^NTR^ restored the LTP deficit and STC impairment that were observed in aging. Furthermore, age‐associated LTD enhancement is counteracted in age‐matched mice without p75^NTR^. These findings indicate that age‐related alterations in synaptic plasticity are not manifested in the absence of p75^NTR^. Together, our data suggest that p75^NTR^ contributes to the detrimental effects of aging on hippocampal long‐term plasticity, associative plasticity, and associative memory.

In our study, we found that the levels of hippocampal p75^NTR^ increased during aging, consistent with previous findings (Costantini et al., 2005; Terry Jr et al., 2011). p75^NTR^ can bind to several different neurotrophins including mature and un‐processed (pro) forms of BDNF. Both proBDNF and mature BDNF regulate synaptic plasticity with opposing effects (Lu et al., 2005). The general consensus in the field is that mature BDNF interacts with the TrkB receptor to promote LTP (Minichiello, 2009; Zagrebelsky et al., 2020), while proBDNF binds to p75^NTR^ and to facilitate LTD (Rösch et al., 2005; Woo et al., 2005). Our data show that the levels of proBDNF increase, while mature BDNF levels remained unchanged in the hippocampus from aged WT mice but not age‐matched p75^NTR^ KO mice. A previous study has suggested that aging affects the processing of proBDNF to the mature form (Buhusi et al., 2017), but the actual mechanisms by which aging increases the levels of proBDNF in hippocampus are unknown. ProBDNF is also a potent inducer of neuronal death (Lee et al., 2001; Nykjaer & Willnow, 2012). Our results demonstrate that the level of hippocampal proBDNF increases in aging, which is believed to contribute to neuronal apoptosis. Interestingly, deletion of p75^NTR^ attenuated age‐dependent increase in the expression of proBDNF and hence, neuronal death in hippocampus.

The MAPK members ERK and p38 are known to mediate opposing forms of long‐term plasticity. Activation of ERK is necessary for LTP, while p38 mediates the induction of LTD (Bolshakov et al., 2000; Huang et al., 2004; Moult et al., 2008). In our study, we found that aging decreased ERK phosphorylation while increased p38 phosphorylation in the hippocampus. How p75^NTR^ connects to members of the MAPK family is not clear. One report had indicated that p75^NTR^ interacts directly with p38 (Wang et al., 2000), but this has not been followed up or verified by other researchers. If correct, it could be envisioned that an age‐related increase in p75^NTR^ level leads to the activation of this MAPK, which is known to regulate remodeling of the actin cytoskeleton (Ronkina et al., 2010). We speculate that, at least in part through p75^NTR^, aging increases p38 activity and negatively modulates MAPK pathways, thus impairing synaptic plasticity and cognitive function.

Activity‐dependent long‐term plasticity is strongly associated with actin proteins, which influence the size and shape of dendritic spines (Rudy, 2015). Our data show that the levels of hippocampal Arc/Arg3.1 decreased in aging, consistent with earlier findings (Ménard & Quirion, 2012; Penner et al., 2011). A decrease in Arc/Arg3.1 has been linked to increased DNA methylation of its promoter region in the CA1 area of hippocampus (Penner et al., 2011). A number of studies have demonstrated that both ERK and p38 modulate the activation of Arc/Arg3.1 transcription (Corrêa et al., 2012; Ronkina et al., 2010; Steward & Worley, 2001). After transcription, Arc/Arg3.1 mRNA is rapidly transported to dendrites and localized to the active dendritic regions (Steward et al., 1998), which promotes the maintenance of LTP and LTM consolidation through regulation of actin dynamics (Bramham & Messaoudi, 2005; Lyford et al., 1995; Steward & Worley, 2001). Taken together, aging reduces synaptic efficacy in hippocampus by decreasing the activation of Arc/Arg3.1.

RhoA‐ROCK‐LIMK‐cofilin pathway has also been implicated in regulating actin dynamics (Tönges et al., 2011). p75^NTR^ interacts with RhoGDI and activates RhoA activity (Yamashita & Tohyama, 2003). Activated RhoA in turn causes ROCK2 activation (Maekawa et al., 1999). In our study, we found that the levels of hippocampal RhoA and ROCK2 are increased in aging, in agreement with a previous finding (VanGuilder Starkey et al., 2013). However, no changes in the expression and activity of RhoA in aged rat hippocampus have also been reported (Kang et al., 2013; Li et al., 2017). RhoA‐ROCK activation activates downstream effectors that regulate cytoskeleton reorganization, such as growth cone collapse and neurite outgrowth inhibition (Lingor et al., 2007; Wu et al., 2013). One of the best characterized downstream targets of ROCK2 is LIMK1 in which its activation results in growth cone collapse (Maekawa et al., 1999). Our data show that aging decreases phosphorylation of hippocampal LIMK1. Modulation of cofilin activity has been shown to be essential for the reorganization of the actin cytoskeleton (Bamburg & Bernstein, 2010). In our study, aging decreases hippocampal cofilin phosphorylation. A study has suggested that p38 activity may contribute to the alteration in cofilin activity in the hippocampus (Eales et al., 2014), corroborating the suggested mechanism that mGluR‐LTD is associated with cytoskeleton reorganization resulting in spine morphological changes, which impact on functional plasticity. Interestingly, we found that deletion of p75^NTR^ prevents the age‐mediated modulation of RhoA‐ROCK2‐LIMK1‐cofilin pathway in the hippocampus. In addition, upregulation of p75^NTR^ is known to be accompanied by the activation of the second messenger ceramide, stabilization of β‐site amyloid precursor protein‐cleaving enzyme‐1 (BACE1) and increased production of amyloid β‐peptide (Aβ) in an age‐dependent fashion (Costantini et al., 2005, 2006).

The p75^NTR^ is also expressed on astrocytes (Dougherty & Milner, 1999; Hanbury et al., 2002; Rudge et al., 1994). In this study, aging was found to increase the expression of p75^NTR^ in astrocytes accompanied with a higher gliosis in the CA1 region of the hippocampus. Interestingly, deletion of p75^NTR^ attenuated age‐dependent change in hippocampal gliosis. A strong induction of p75^NTR^ has been observed on astrocytes after different types of injury (Hanbury et al., 2002; Oderfeld‐Nowak et al., 2003) although the role of this receptor in these pathological conditions remains enigmatic. Astrocytes release glutamates, which are particularly important in regulating synaptic plasticity and memory (Fields & Stevens‐Graham, 2002; Ota et al., 2013). However, the possible implications of the astrocytes with increased p75^NTR^ in hippocampus in mediating age‐associated changes in synaptic plasticity and memory will need to be explored further in future.

**FIGURE 12:**
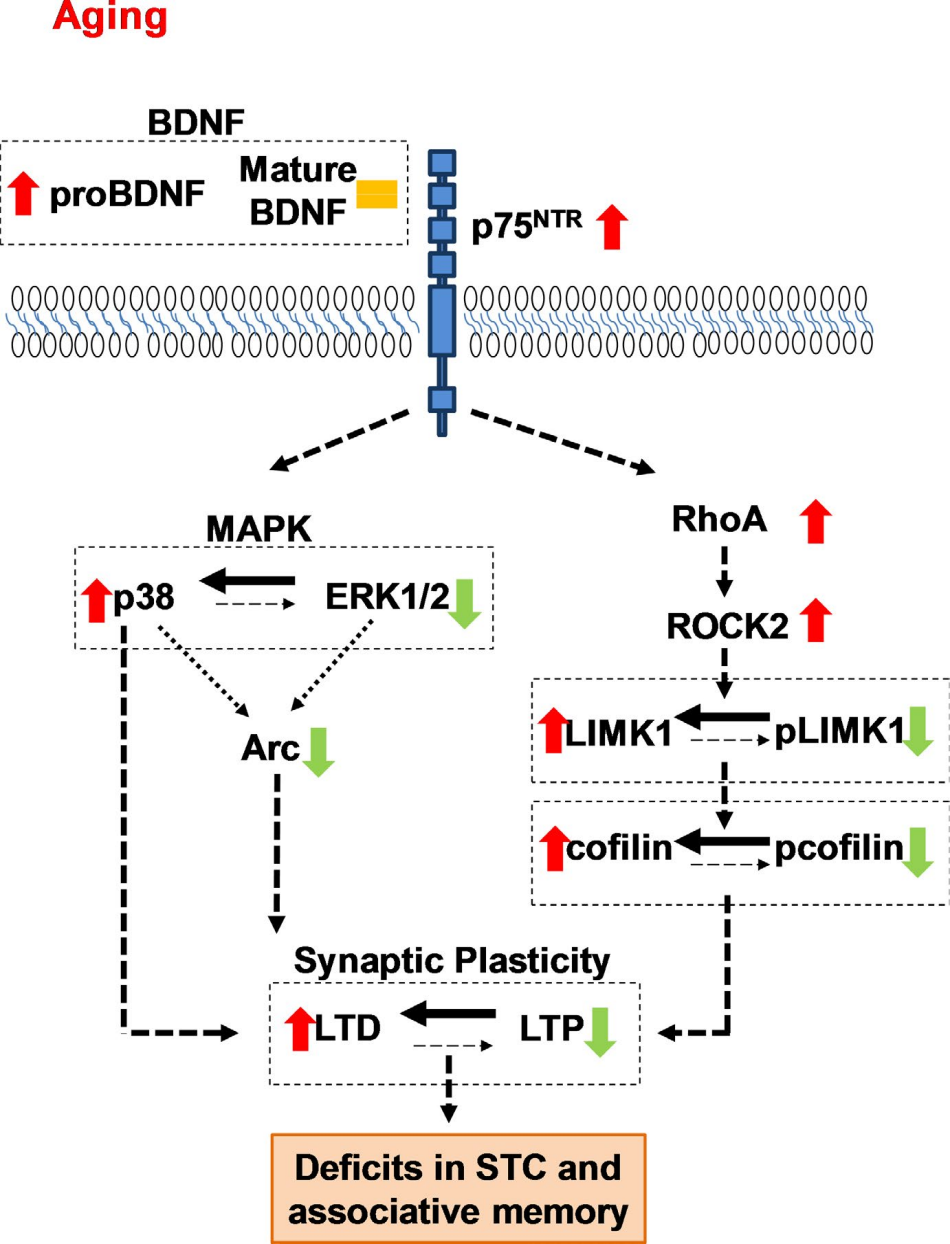
The molecular mechanism of p75^NTR^ in regulating age‐associated changes in the homeostatic of synaptic plasticity. This cartoon depicts the signaling pathway by p75^NTR^ in mediating synaptic plasticity changes in aging. Aging increases proBDNF without affecting mature BDNF. ProBDNF has been implicated in facilitating LTD. Aging also modulates MAPK pathway by upregulating p38 activity while downregulating ERK1/2 activity. Both p38 and ERK1/2 pathways are important in regulating Arc gene transcription. Aging decreases Arc protein and thus, affecting the maintenance of LTP and LTM consolidation through regulation of actin dynamics. In addition, aging increases RhoA level leading to an increase in ROCK2 activity. This reduces both LIMK1 and cofilin phosphorylation. Modulation of cofilin activity is essential for the reorganization of the actin cytoskeleton and influences synaptic plasticity. As a whole, p75^NTR^ responsible for the age‐mediated disruption of hippocampal homeostatic long‐term plasticity by modulating several signaling pathways, including BDNF, MAPK, Arc, and RhoA‐ROCK2‐LIMK1‐cofilin, leading to deficits in STC and associative memory. Red arrow indicates increases. Green arrow indicates decreases. Orange equals sign indicates no change

In conclusion, our findings demonstrate that p75^NTR^ is an important mediator for the alterations in hippocampal‐dependent synaptic plasticity and memory caused by aging. In general, deletion of p75^NTR^ prevents age‐mediated changes in several important pathways affecting synaptic plasticity and behavior, including BDNF, MAPK, Arc, and RhoA‐ROCK2‐LIMK1‐cofilin (Figure 12). Alterations in these pathways underlie the disruption of homeostatic plasticity in hippocampus, which eventually contributes to the failure of long‐term plasticity and associative plasticity, and hence associative memory in aging. Thus, targeting p75^NTR^ could be an attractive strategy to counteract the impairments in cognitive function that result from aging.

## 4 MATERIALS AND METHODS

### 4.1 Animals

In this study, a total of 29 male C57BL/6J 5–7 weeks, 28 male p75^NTR^ KO 5–7 weeks mice, 32 male C57BL/BJ 22–24 months, and 34 male p75^NTR^ KO 22–24 months mice were used for the experiments. p75^NTR^ KO mice (exon III; Lee et al., 1992) were obtained from the Jackson Laboratory and back crossed to the C57BL/6 J background for at least 10 generations. Animals were housed under 12‐h light/dark cycle in standard cages with food and water *ad libitum*. All experimental procedures using animals were carried out in accordance with protocols approved by the Institutional Animal Care and Use Committees (IACUC) at the National University of Singapore.

### 4.2 Electrophysiology

The mice were anesthetized using CO_2_ and decapitated. The brains were quickly removed and cooled in 4°C artificial cerebrospinal fluid (ACSF). For most of the experiments, the ACSF contained the following (in millimolars): 124 NaCl, 3.7 KCl, 1 MgSO_4_.7H_2_O, 2.5 CaCl_2_.2H_2_O, 1.2 KH_2_PO_4_, 24.6 NaHCO_3_, and 10 D‐glucose. For metabotropic glutamate receptor (mGluR)‐LTD experiments, the ACSF contained the following (in millimolars): 124 NaCl, 5 KCl, 1 MgSO_4_.7H_2_O, 2 CaCl_2_, 1.25 NaH_2_PO_4_, 26 NaHCO_3_, and 10 D‐glucose. The ACSF was equilibrated with 95% O_2_–5% CO_2_ (carbogen; total consumption 16 L/h). Transverse hippocampal slices (400 μm thick) were prepared from the hippocampus by using a manual tissue chopper. The hippocampal CA3 region was removed for mGluR‐LTD experiments. The slices were then incubated at 32°C in an interface chamber (Scientific System Design) at an ACSF flow rate of 1 ml/min. Two‐pathway experiments were performed for most of the experiments. Two monopolar, lacquer‐coated, stainless‐steel electrodes (5 MΩ; AM Systems) were positioned at an adequate distance within the stratum radiatum of the CA1 region for stimulating two independent synaptic inputs S1 and S2 of one neuronal population, thus evoking field excitatory post‐synaptic potential (fEPSP) from Schaffer collateral‐commissural‐CA1 synapses. For mGluR‐LTD recordings, single‐pathway experiments were performed. One monopolar, lacquer‐coated, stainless‐steel electrode was positioned within the stratum radiatum of the CA1 region for stimulating synaptic input of the neuronal population, thus evoking fEPSP from Schaffer collateral‐commissural‐CA1 synapses. For recording, one electrode was placed in the CA1 apical dendritic layer. The signals were amplified by a differential amplifier (Model 1700, AM Systems) and digitized using a CED 1401 analog‐to‐digital converter (Cambridge Electronic Design). After the pre‐incubation period of 3 hours, an input–output curve (afferent stimulation vs fEPSP slope) was plotted prior to experiments. To set the test stimulus intensity, a fEPSP of 40% of its maximal amplitude was determined. Biphasic constant‐current pulses were used for stimulation. To evaluate basal excitatory synaptic transmission, a series of pulses of microamperes were applied to generate an input–output curve. Results were presented as stimulus intensity versus fEPSP slope. The short‐term synaptic modification was measured by pair‐pulse facilitation (PPF) (Arias‐Cavieres et al., 2017). PPF was evoked with interstimulus intervals of 50 ms at submaximal stimulus intensities. PPF was expressed as the ratio of the fEPSP slope (second stimulation)/fEPSP slope (first stimulation) (Arias‐Cavieres et al., 2017). Late‐LTP was induced using three stimulus trains of 100 pulses strong tetanization (STET), 100 Hz; duration, 0.2 ms/polarity; and inter‐train interval, 10 min. A weak tetanization (WTET) protocol consisting of one 100 Hz train (21 biphasic constant‐current pulses; pulse duration per half‐wave, 0.2 ms) was used to induce early‐LTP. In experiments in which early‐LTD was investigated, a weak low‐frequency stimulation (WLFS) protocol was used consisting of 900 pulses at a frequency of 1 Hz, an impulse duration of 0.2 ms/half‐wave, with 900 total stimuli. mGluR‐LTD was chemically induced using group I mGluR agonist (S)‐3,5‐dihydroxyphenylglycine (DHPG; Tocris). The final concentrations used for DHPG were 100 µM. The slopes of fEPSPs were monitored online. The baseline was recorded for 30 min. For baseline recording and testing at each time point, four 0.2 Hz biphasic constant‐current pulses (0.1 ms/polarity) were used (Shetty et al., 2015).

### 4.3 Western Blotting

Whole hippocampi were isolated and snap frozen in liquid nitrogen and stored at −80°C. The protein extraction was performed by using Tissue Protein Extraction reagent (T‐PER; Thermo Scientific, USA) supplemented with protease and phosphatase inhibitor (Thermo Scientific, USA) according to the manufacturer's protocol, followed by centrifugation for 5 min at 10,000 rpm at 4°C. The protein concentration was determined using the Bradford assay (Bio‐Rad). Appropriate protein concentration was added to the sample buffer and heated at 95°C for 10 min before separated by SDS‐polyacrylamide gels. Gels were transferred to PVDF membranes (Bio‐Rad) in a semi‐dry transfer cell (Bio‐Rad) for 1 hour at 15 V. The membranes were blocked with 5% w/v nonfat dry milk or bovine serum albumin in 1X TBST. The membranes were then immunoblotted with primary antibodies. The primary antibodies with their concentrations used were as follows: goat anti‐p75^NTR^ (1:1000, Neuromics), rabbit anti‐BDNF (1:1000, Abcam), rabbit anti‐NGF (1:1000, Abcam), rabbit anti‐total or phospho‐p38 (1:1000; Cell Signaling), rabbit anti‐total or phospho‐ERK (1:1000; Cell Signaling), rabbit anti‐RhoA (1:1000, Abcam), rabbit anti‐ROCK2 (1:1000; Cell Signaling), total anti‐LIMK1 (1:1000; Cell Signaling), rabbit anti‐phospho‐LIMK1 (1:1000; Abcam), rabbit anti‐total or phospho‐cofilin (1:1000, Cell Signaling), and mouse anti‐tubulin (1:20,000; Sigma‐Aldrich). The membranes were incubated with the secondary peroxidase‐conjugated antibodies. Signals were generated by using the SuperSignal® West Pico Chemiluminescent Substrate (Thermo Scientific, USA). The amounts of all the proteins were quantified by densitometric measurement of Western blots using ImageJ (NIH software). The densitometric values of each blot were normalized to the amounts of tubulin, which served as loading control and were calculated in relation to the control group.

### 4.4 Immunostaining

Mice were perfused first with PBS, followed by 4% paraformaldehyde. Harvested brains were post‐fixed in 4% paraformaldehyde for 16 hours and cryoprotected in 30% sucrose before freezing. OCT‐embedded brains were frozen at −80°C overnight and serially sectioned at 30 μm in the coronal plane using cryostat. Sections were mounted onto electrostatic charged slides (Leica Microsystems), blocked with 5% donkey serum (Fisher scientific) containing 0.3% Triton X‐100 (Sigma‐Aldrich) in PBS for 1 hour at room temperature, and then incubated for 16 hours at 4 °C with primary antibodies. The sections were washed in PBS before incubated with the appropriate secondary antibodies. The primary antibodies used in this study were as follows: anti‐p75^NTR^ (1:500; Neuromics), anti‐MAP2 (1:2000; Abcam), anti‐GFAP (1:500; Abcam), anti‐Iba1 (1:200; Wako), and anti‐NeuN (1:200; Millipore). The secondary antibodies used in this study were as follows: donkey anti‐goat IgG Alexa Fluor 555 (1:1000; Invitrogen), donkey anti‐rabbit IgG Alexa Fluor 647 (1:1000; Invitrogen), donkey anti‐mouse IgG Alexa Fluor 647 (1:1000; Invitrogen), and donkey anti‐chicken IgG Alexa Fluor 488 (1:1000; Sigma‐Aldrich). For TUNEL staining, the sections were stained as per instruction provided in TUNEL kit (Click‐iT^TM^ plus TUNEL Assay for In Situ Apoptosis Detection, Alexa Fluor^TM^ 488 dye). Images were obtained using a Zeiss Axioplan confocal microscope.

### 4.5 RhoA activation assay

Protein was extracted from mice hippocampi. RhoA activity was evaluated in total hippocampal extracts using the RhoA G‐Lisa kit (Cytoskeleton, BK124) following the manufacturer's instructions. Equal amount of protein was used from each sample as determined by Bradford Assay.

### 4.6 Behavioral tagging study

In this study, the mice were placed in an open field (OF) for 10 min within 1 hour before weak inhibitory avoidance (IA) training. The OF is a plastic box, and the IA apparatus consists of a Plexiglas box with a platform on the left end of a series of bars, which constituted the floor of the box. Mice were placed on the platform facing the left rear corner of the box during the training session. They received a weak foot shock (0.3 mA, 2 s) when they stepped down and put four paws on the bars. The mice were then removed from the box and returned to their home cage. Memory was measured by comparing the step‐down latency in the training session to that in the test session. The cut‐off time for step‐down latency was 4 min. The higher percentage of step‐down latency indicates animals have better associative memory. Memory was tested at 1 h, 24 h, and 7 days after training sessions. More details about the procedures are described in (Wong et al., 2019, 2020).

### 4.7 Statistical analysis

All data are represented as mean ± SEM. The fEPSP slope value expressed as percentages of average baseline values per time point was subjected to statistical analysis using GraphPad Prism 6.0 (GraphPad, San Diego, CA, USA). Nonparametric tests were used as the normality variations at small sample sizes. Wilcoxon signed rank test (Wilcox test) was used to compare within one group and Mann‐Whitney *U* test (*U* test) was used when data were compared between groups. Statistical comparisons for the Western blot and behavioral tagging experiments were performed using two‐way ANOVA with Tukey's post hoc test. *p* < 0.05 was considered as the cutoff for statistically significant difference.

## CONFLICT OF INTEREST

The authors declare no conflict of interests.

## AUTHOR CONTRIBUTIONS

L.‐W.W, C.F.I, and S.S designed research; L.‐W.W, Y.S.C, and W.L performed electrophysiology, biochemical, and behavioral experiments; L.K and E.S performed immunostaining and image analysis. L.‐W.W and S.S analyzed data and wrote the paper. C.F.I provided valuable comments and discussion.

## Supporting information


**Figure S1**

**Figure S2**

**Figure S3**

## Data Availability

The data that support the findings of this study are available upon request.
