# Supplementary figures and images for: Age‐related changes in hippocampal‐dependent synaptic plasticity and memory mediated by p75 neurotrophin receptor
Source: Aging Cell. 2021 Jan 15;20(2):e13305. doi: 10.1111/acel.13305 (PMC7884039; doi:10.1111/acel.13305)

**Figure S1**

**A**

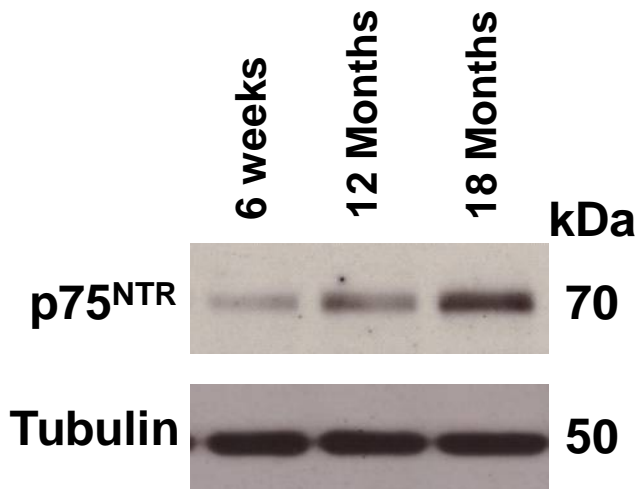

**B**

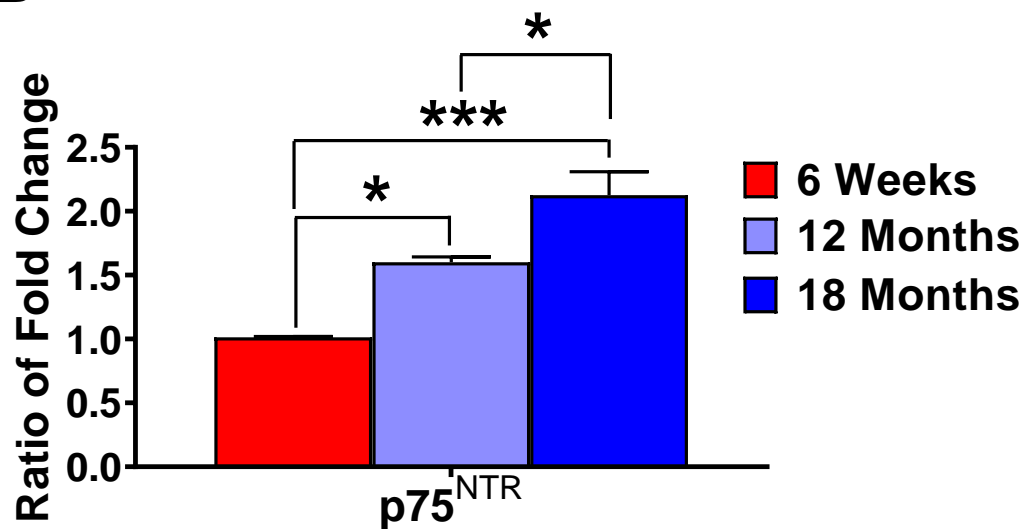

Supplement: Supplementary file 1 — Figure S1 [file ACEL-20-e13305-s001.pdf]

Figure S2

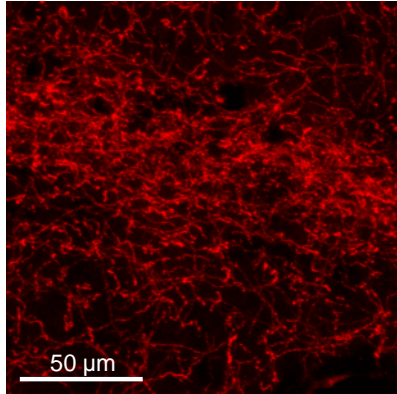

p75<sup>NTR</sup>

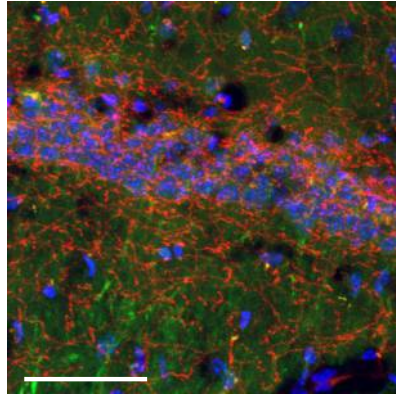

p75<sup>NTR</sup>, MAP2, DAPI

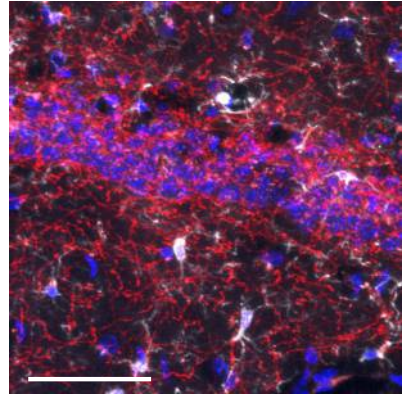

p75<sup>NTR</sup>, Iba1, DAPI

Supplement: Supplementary file 2 — Figure S2 [file ACEL-20-e13305-s002.pdf]

**Figure S3**

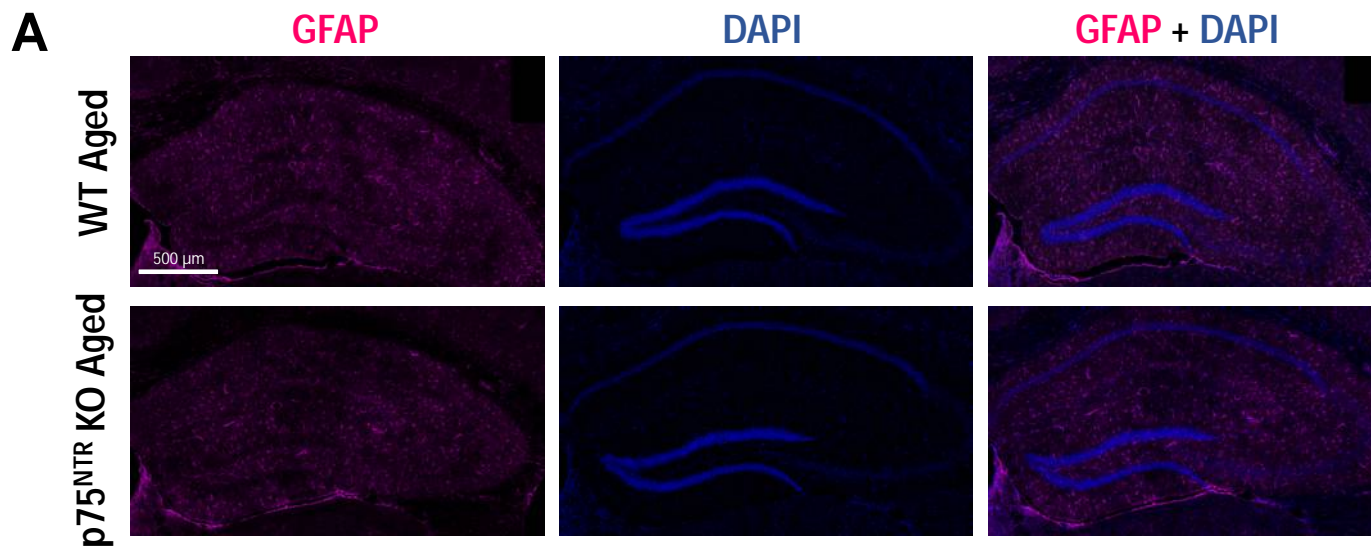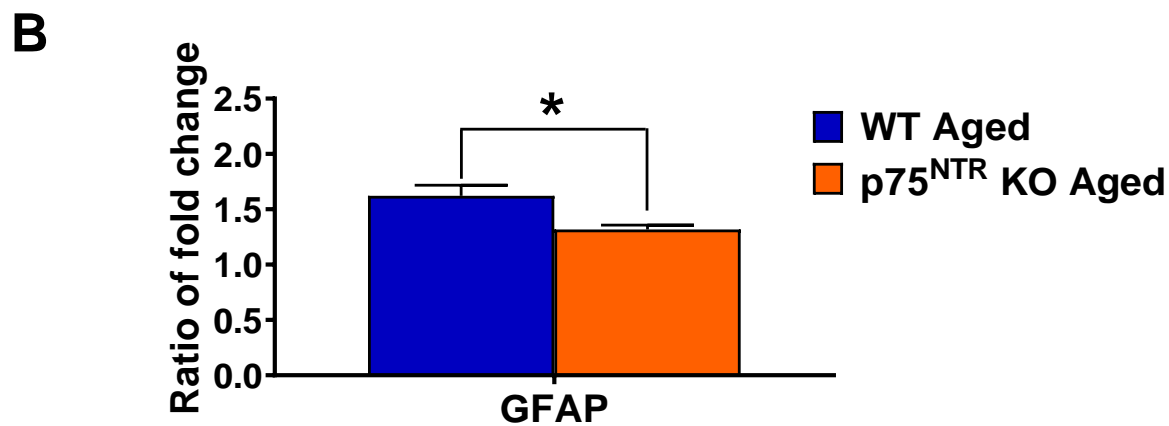

Supplement: Supplementary file 3 — Figure S3 [file ACEL-20-e13305-s003.pdf]
